# Mycotoxin exposure following an eight-week vegan or meat-rich dietary intervention: a randomized-controlled trial

**DOI:** 10.1038/s41538-026-01152-4

**Published:** 2026-09-18

**Authors:** Pia Sophie Kanonier, Stefanie Kowarschik, Roman Huber, Maximilian Andreas Storz

**Affiliations:** Department of Internal Medicine II, Centre for Complementary Medicine, Medical Center – University of Freiburg, Faculty of Medicine, University of Freiburg, Freiburg, Germany

**Keywords:** Diseases, Environmental sciences, Health care, Microbiology, Risk factors

## Abstract

Mycotoxin exposure in humans has important food safety implications. We analyzed crude and creatinine-adjusted mycotoxin excretion in urine samples obtained from a two-arm, randomized-controlled trial in healthy adults. *N* = 63 participants were allocated to a vegan diet (VD) or a meat-rich diet (MD) for eight weeks. After the dietary intervention (DI), median deoxynivalenol concentrations were higher in the VD group (7.15 (IQR: 8.10) *vs*. 2.85 (IQR: 6.04) µg/g in the MD group, *p* = 0.004) whereas median T-2 toxin concentrations were higher in the MD group (5.15 (IQR: 5.81) vs. 3.44 (IQR: 3.67) µg/g in the VD group, *p* = 0.047). Higher creatinine-adjusted ochratoxin A (0.45 (IQR: 0.37) vs. 0.20 (IQR: 0.28) µg/g) and zearalenone concentrations (1.34 (IQR: 1.52) vs. 0.49 (IQR: 1.01) µg/g) were observed in the VD group (*p* = 0.003 and 0.001, respectively). For deoxynivalenol, ochratoxin A and zearalenone, mixed model repeated measures pointed in the same direction. Mycotoxin exposure for vegans differed from omnivores subsequent to an isocaloric DI and requires attention from a food safety perspective.

## Introduction

Most filamentous fungi produce secondary metabolites known as mycotoxins^1,2^. More than 1000 distinct mycotoxins have been recognized so far, with aflatoxins, ochratoxins, fumonisins, patulin, zearalenone, nivalenol, deoxynivalenol and ergot alkaloids being the most common mycotoxins of concern for humans and livestock^1,3,4^. Recent reviews suggested that up to 80% of the world’s food crops are contaminated by mycotoxins, which poses a huge risk for human exposure^5,6^.

Mycotoxin exposure in humans has important public health and food safety implications and has been linked to carcinogenicity, nephrotoxicity, hepatotoxicity, immunosuppression, and estrogenic disruption^4,7–9^.

Plant-based foods, such as cereals, grains, fruits, nuts and seeds as well as coffee and spices are traditionally among the major mycotoxin sources in the human diet^10,11^. In addition, plant-based meat-, cheese-, and fish-alternatives are now considered novel, emerging mycotoxin sources, with seitan-based products and plant-based milk alternatives reported as particularly high in certain mycotoxins^12,13^. This may render individuals who choose a plant-based (vegetarian or vegan) diet particularly vulnerable to an increased mycotoxin exposure and potentially adverse health outcomes^14–21^.

Several studies have recently assessed mycotoxin exposure and excretion in vegetarians and vegans in comparison to omnivores^16–18^. Notably, these studies often revealed conflicting results. While some studies suggested a higher exposure to mycotoxins with a vegan diet^16^ or a vegetarian diet^18^, others did not^17^. In addition, mycotoxin exposure assessment was not homogenous across studies with specific mycotoxins highlighted and examined in each study (e.g., deoxynivalenol by Wells et al. in the UK or ochratoxin A by Penczynski et al. in Germany)^16,18^.

To the best of our knowledge, there are no large randomized-controlled trials in this emerging field. Most studies conducted in vegans in the past had a cross-sectional design and did not fully control for external confounders such as diet duration. To address this gap in the literature, we revisited a previously conducted randomized-controlled trial with vegans and omnivorous and determined six mycotoxins in the urine of *n* = 63 study participants who either completed an isocaloric eight-week vegan or meat-rich dietary intervention^22,23^. All study participants were omnivores and reported a mixed diet upon recruitment. Based on the previous literature from Germany^16^, we hypothesized that vegans would be exposed to a higher mycotoxin burden subsequent to the dietary intervention and the accompanying increased plant food intake. As a secondary outcome, we sought to identify potential food sources associated with an increased mycotoxin intake.

## Results

The final study sample included *n* = 63 participants. Supplementary Fig. 2 displays a study-specific inclusion flowchart with reasons for in- and exclusion of participants. Table 1 displays the sample characteristics. No relevant differences in baseline characteristics were observed when comparing individuals assigned to the VD and MD, respectively.

**Table. 1:** Sample characteristics at baseline (week 0) and after the dietary intervention (week 8) by diet group (vegan diet (VD) vs. meat-rich diet (MD))

Variable	Full sample (*n* = 63)	VD group (*n* = 32)	MD group (*n* = 31)	*p*-value
*Baseline (week 0)*				
**Sex**				
Male	*n* = 37 (58.73%)	*n* = 19 (59.38%)	*n* = 18 (58.06%)	0.916^c^
Female	*n* = 26 (41.27%)	*n* = 13 (40.63%)	*n* = 13 (41.94%)	
**Age (years)**	23 (3)	22 (2.5)	24 (3)	0.227^d^
**Weight (kg)**	73.68 ± 9.83^a^	73.72 ± 9.48^b^	73.64 ± 10.31	0.975^e^
**Height (cm)**	176.18 ± 8.95^a^	177.05 ± 9.13^b^	175.31 ± 8.83	0.448^e^
**BMI (kg/m**^**2**^**)**	23.36 (2.98)^a^	23.53 (2.67)^b^	23.35 (3.24)	0.476 ^d^
**MASSS**	6.86 ± 1.22	6.84 ± 1.32	6.87 ± 1.12	0.930^e^
**Physical activity level**				
Inactive	*n* = 2 (3.17%)	*n* = 2 (6.25%)	*n* = 0 (0%)	0.537^f^
Minimally active	*n* = 23 (36.51%)	*n* = 12 (37.50%)	*n* = 11 (35.48%)	
HEPA active	*n* = 38 (60.32%)	*n* = 18 (56.25%)	*n* = 20 (64.52%)	
**Highest education level**				
German “Abitur”	*n* = 49 (77.77%)	*n* = 28 (87.50%)	*n* = 21 (67.74%)	0.059^c^
University degree	*n* = 14 (22.22%)	*n* = 4 (12.50%)	*n* = 10 (32.26%)	
*Week 8*				
**Weight (kg)**	72.43 ± 9.75	72.38 ± 4.49	72.49 ± 10.17	0.963^e^
**Height (cm)**	176.74 ± 8.76	177.77 ± 8.84	175.69 ± 8.69	0.351^e^
**BMI (kg/m**^**2**^**)**	22.77 (2.92)*	22.62 (2.54)*	22.96 (3.27)*	0.306^d^
**MASSS**	6.98 ± 1.35	6.97 ± 1.40	7.00 ± 1.32	0.928^e^
**Physical activity level**				
Inactive	*n* = 1 (1.59%)	*n* = 1 (3.13%)	*n* = 0 (0%)	0.348^f^
Minimally active	*n* = 20 (31.74%)	*n* = 8 (25.00%)	*n* = 12 (38.71%)	
HEPA active	*n* = 42 (66.67%)	*n* = 23 (71.88%)	*n* = 19 (61.29%)	

Continuous data displayed with their mean ± standard deviation if normally distributed or as median (interquartile range) if not normally distributed. Categorical data displayed as *n* = x (%).

*MASSS* MacArthur Scale of Subjective Social Status,* HEPA* Health Enhancing Physical Activity.

^a^ Based on *n* = 62 observations.

^b ^Based on *n* = 31 observations (no anthropometric data available for one person at baseline in the VD group).

^c^ Based on Chi-Square analyses.

^d^ Based on Wilcoxon (Mann–Whitney) rank sum test analyses.

^e^ Based on Student’s *t* test analyses.

^f^ Based on Fisher’s exact test.

*indicates significant differences in comparison to baseline at a *p* < 0.001.

Tables 2 and 3 show crude and creatinine-adjusted urinary mycotoxin concentrations at baseline and after eight weeks into the study in the full sample and in a diet-specific manner. Relevant between-group differences at week 8 were observed for urinary creatinine-adjusted deoxynivalenol concentrations (with a *p* value of 0.004) and T-2 toxin concentrations (with a *p* value of 0.047). Deoxynivalenol concentrations were higher in the VD group (7.15 (8.10) µg/g vs. 2.85 (6.04) µg/g in the MD group) whereas T-2 toxin concentrations (in µg/g) were higher in the MD group (5.15 (5.81) vs. 3.44 (3.67) in the VD group). Higher creatinine-adjusted ochratoxin A concentrations (0.45 (0.37) vs. 0.20 (0.28) µg/g) and zearalenone concentrations (1.34 (1.52) *vs*. 0.49 (1.01) µg/g) were observed in the VD group at week 8 (with *p* values of 0.003 and 0.001, respectively).

**Table 2:** Crude mycotoxin concentrations in urine at baseline (week 0) and after eight weeks of the dietary intervention

Crude mycotoxin concentrations	Full sample (*n* = 63)			VD group (*n* = 32)			MD group (*n* = 31)			Mann–Whitney U test	
	Week 0	Week 8	*p*-value (w0 vs. w8)	Week 0	Week 8	*p*-value (w0 vs. w8)	Week 0	Week 8	*p*-value (w0 vs. w8)	*p-*value (w0)	*p*-value (w8)
Aflatoxin (µg/L)	0.11 (0.00)	0.11 (0.00)	0.566^a^	0.11 (0.00)	0.11 (0.00)	0.281^a^	0.11 (0.00)	0.11 (0.00)	**0.031**^a^	0.170	0.088
Deoxynivalenol (µg/L)	3.49 (4.01)	3.54 (4.81)	0.586^a^	3.50 (3.84)	3.66 (5.28)	0.314^a^	3.19 (4.01)	2.79 (4.05)	0.763^a^	0.929	0.271
Ochratoxin A (µg/L)	0.17 (0.26)	0.24 (0.21)	0.217^a^	0.15 (0.22)	0.24 (0.25)	0.121^a^	0.19 (0.21)	0.24 (0.19)	0.866^a^	0.287	0.649
T-2 toxin (µg/L)	2.87 (4.74)	4.26 (6.82)	0.056^a^	2.78 (4.78)	1.94 (4.35)	0.566^a^	3.67 (4.74)	6.42 (7.36)	**0.001**^a^	0.925	**0.005**
Zearalenone (µg/L)	0.78 (1.12)	0.67 (1.12)	0.474^a^	0.70 (1.16)	0.70 (1.32)	0.413^a^	0.87 (0.74)	0.60 (0.59)	**0.039**^a^	0.335	0.138
Fumonisin (µg/L)	1.78 (0.00)	1.78 (0.00)	0.500^a^	1.78 (0.00)	1.78 (0.00)	1.000^a^	1.78 (0.00)	1.78 (0.00)	1.000^a^	1.000	1.000

Mycotoxin detection limits (d.l.) were as follows: Aflatoxin: 0.21 µg/L; Deoxynivalenol: 2.79 µg/L; Fumonisin: 3.55 µg/L; Ochratoxin A: 0.11 µg/L; T-2 toxin: 1.46 µg/L; Zearalenone: 0.52 µg/L. Measurements below the lower detection limit were set to d.l./2. Data displayed as medians (interquartile range).

*MD* Meat-Rich Diet, *VD* Vegan Diet.

^a^ Based on Wilcoxon signed-rank test.

*P*-values < 0.05 are shown in bold.

**Table 3:** Creatinine-adjusted mycotoxin concentrations in urine at baseline (week 0) and after eight weeks of the dietary intervention

Creatinine-adjusted mycotoxin levels	Full sample (*n* = 63)			VD group (*n* = 32)			MD diet group (*n* = 31)			Mann–Whitney U test		
	Week 0	Week 8	*p*-value (w0 *vs*. w8)	Week 0	Week 8	*p*-value (w0 *vs*. w8)	Week 0	Week 8	*p*-value (w0 *vs*. w8)	*p-*value (w0)	*p*-value (w8)	
Deoxynivalenol (µg/g)	4.36 (8.90)	5.46 (8.13)	0.538^a^	4.13 (8.76)	7.15 (8.10)	0.172^a^	4.36 (7.87)	2.85 (6.04)	0.456^a^	0.601		**0.004**
Ochratoxin A (µg/g)	0.28 (0.26)	0.32 (0.36)	**0.023**^a^	0.26 (0.25)	0.45 (0.37)	**0.001**^a^	0.33 (0.31)	0.20 (0.28)	0.779^a^	0.284		**0.003**
T-2 toxin (µg/g)	3.65 (3.94)	4.59 (4.58)	0.117^a^	3.48 (4.96)	3.44 (3.67)	0.803^a^	3.86 (3.51)	5.15 (5.81)	**0.010**^a^	0.809		**0.047**
Zearalenone (µg/g)	1.19 (1.07)	1.00 (1.65)	0.762^a^	1.23 (1.12)	1.34 (1.52)	0.139^a^	1.08 (0.99)	0.49 (1.01)	**0.022**^a^	0.878		**0.001**

Mycotoxin detection limits (d.l.) were as follows: Deoxynivalenol: 2.79 µg/L; Ochratoxin A: 0.11 µg/L; T-2 toxin: 1.46 µg/L; Zearalenone: 0.52 µg/L. Measurements below the lower detection limits were set to d.l./2. Data displayed as medians (interquartile range).

*MD* Meat-Rich Diet, *VD* Vegan Diet.

^a^ Based on Wilcoxon signed-rank test.

*P*-values < 0.05 are shown in bold.

**Fig. 1:**
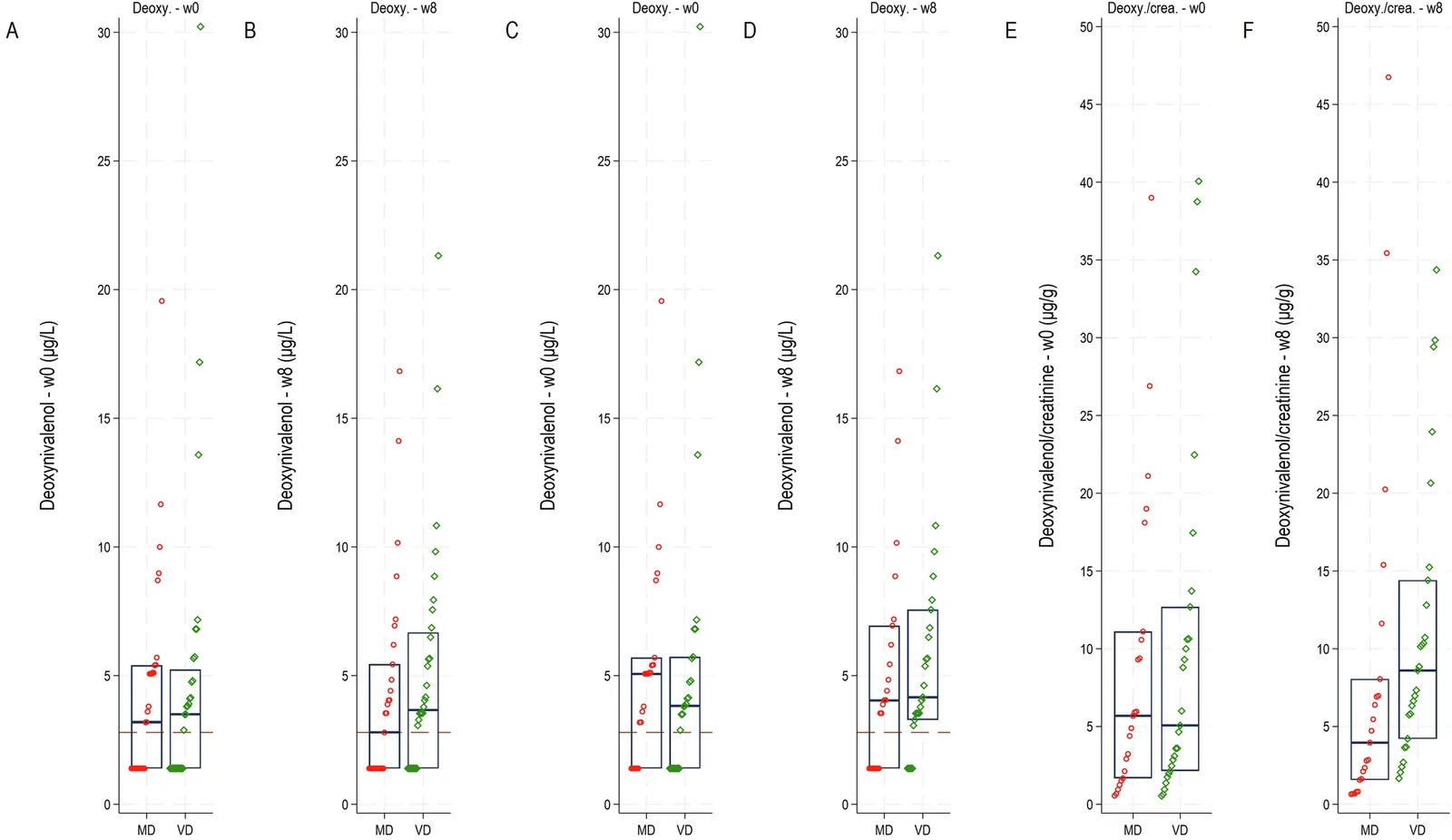
Strip plot displaying deoxynivalenol excretion in urine at baseline (week 0) and after eight weeks into the study with a meat-rich diet (red color) or vegan diet (green color). **A**, **B:** Both panels show the entire sample (including those observations below the detection limit) whereas panels **C**–**F** show only participants above the detection limit (2.79 μg/L). Panels **E**, **F** display creatinine-adjusted values (in μg/g). The horizontal dashed brown line marks the detection limit. MD Meat-Rich Diet, VD Vegan Diet. Based on *n* = 63 paired observations. Deoxynivalenol concentrations were below the detection limit in *n* = 28 samples (44.44%) at baseline and *n* = 25 samples (39.68%) after eight weeks, respectively.

**Fig. 2:**
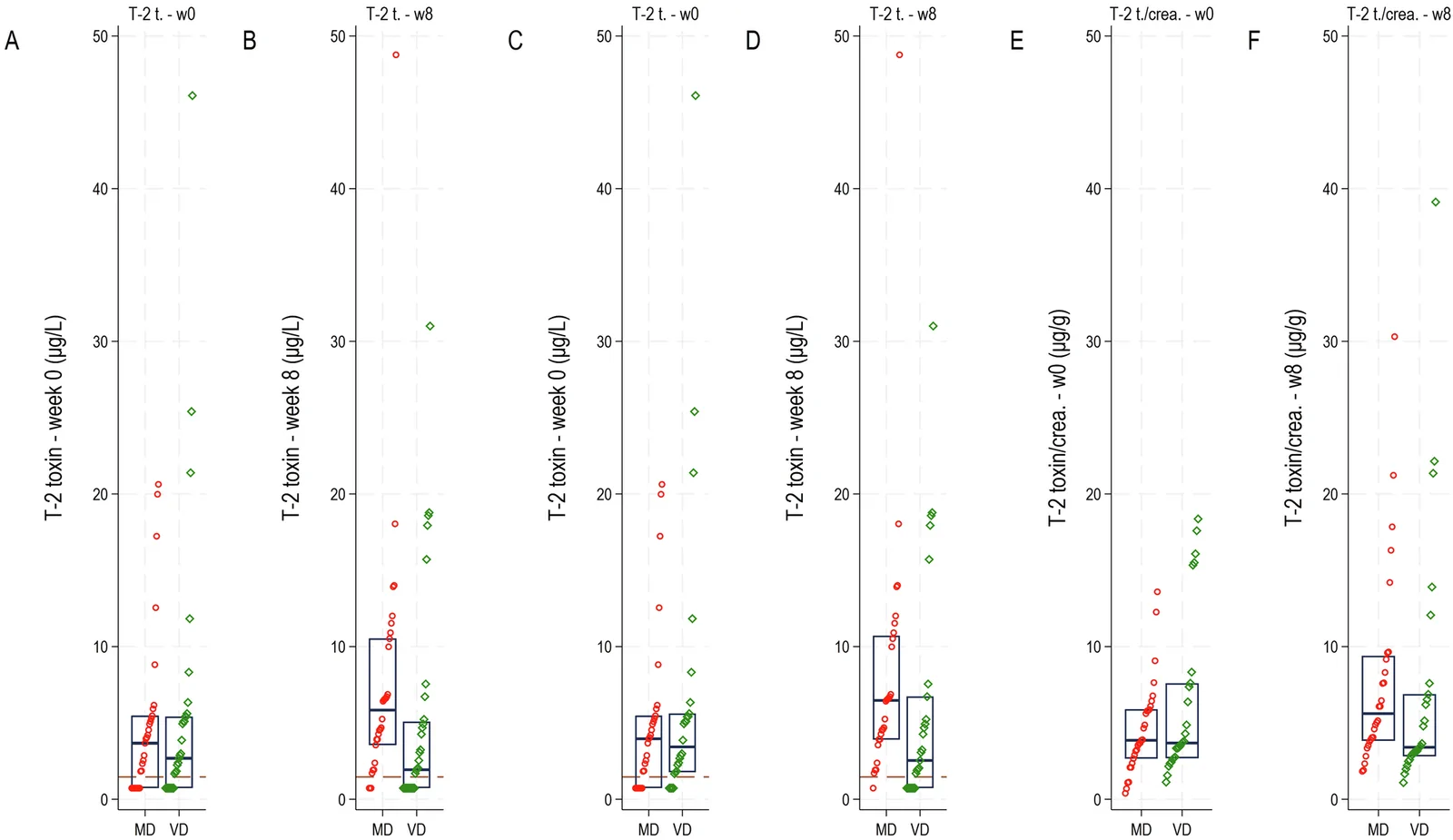
Strip plot displaying T-2 toxin excretion in urine at baseline (week 0) and after eight weeks into the study with a meat-rich diet (red color) or vegan diet (green color). **A**, **B** Both panels show the entire sample (including those observations below the detection limit) whereas panels **C**–**F** show only participants above the detection limit (1.46 μg/L). Panels **E**, **F** display creatinine-adjusted values (in μg/g). The horizontal dashed brown line marks the detection limit. MD Meat-Rich Diet, VD Vegan Diet. Based on *n* = 63 paired observations (one person is not displayed in panels **A**–**F** for exceeding the y-scale limit of 50 μg/L). T-2 toxin concentrations were below the detection limit in *n* = 20 samples (31.75%) at baseline and *n* = 17 samples (26.98%) after eight weeks, respectively.

**Fig. 3:**
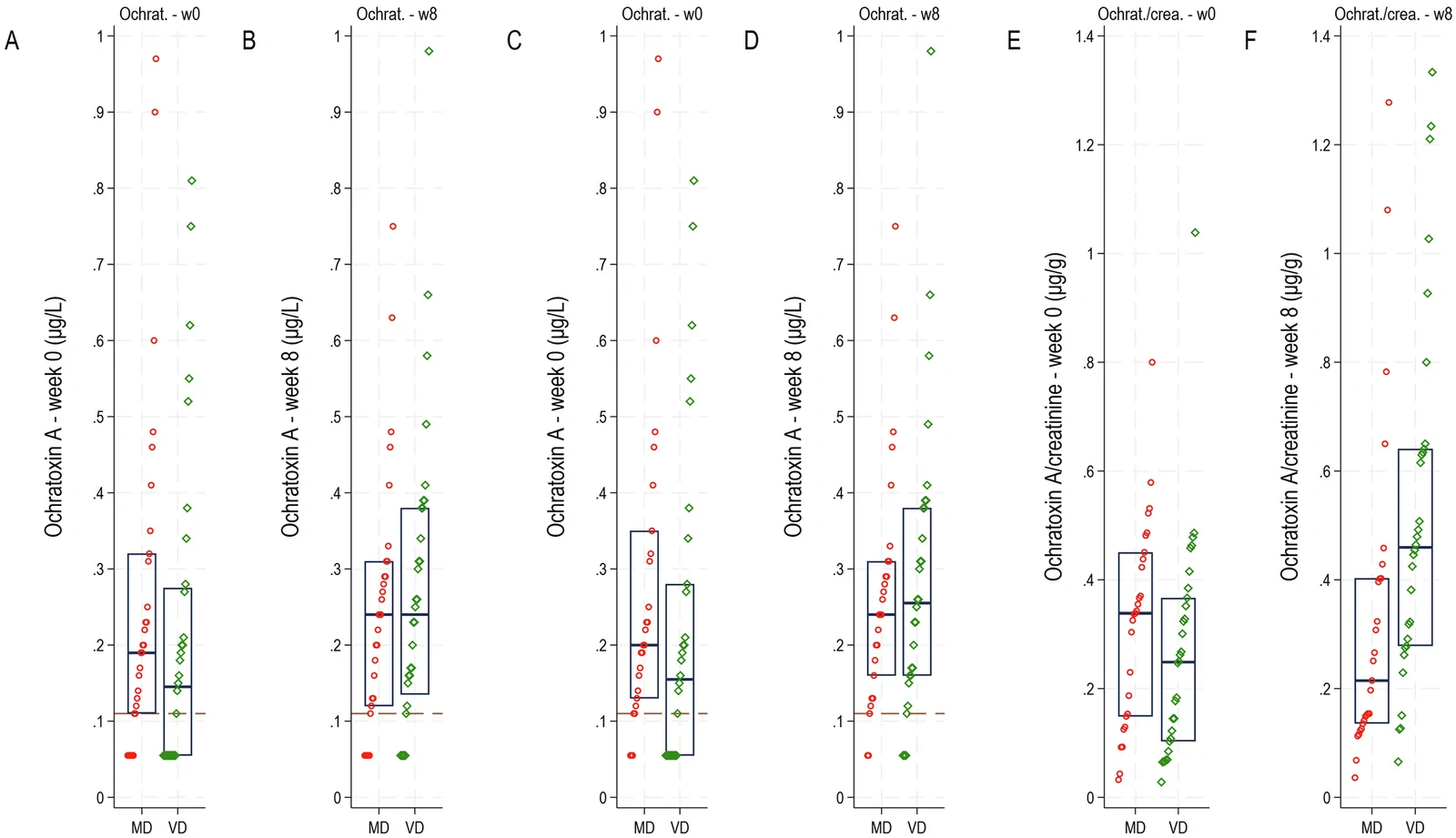
Strip plot displaying ochratoxin A excretion in urine at baseline (week 0) and after eight weeks into the study with a meat-rich diet (red color) or vegan diet (green color). **A**, **B** Both panels show the entire sample (including those observations below the detection limit) whereas panels **C**–**F** show only participants above the detection limit (0.11 μg/L). Panels **E**, **F** display creatinine-adjusted values (in μg/g). The horizontal dashed brown line marks the detection limit. MD Meat-Rich Diet, VD Vegan Diet. Based on *n* = 63 paired observations. Ochratoxin A concentrations were below the detection limit in *n* = 21 samples (33.33%) at baseline and *n* = 12 samples (19.05%) after eight weeks, respectively.

**Fig. 4:**
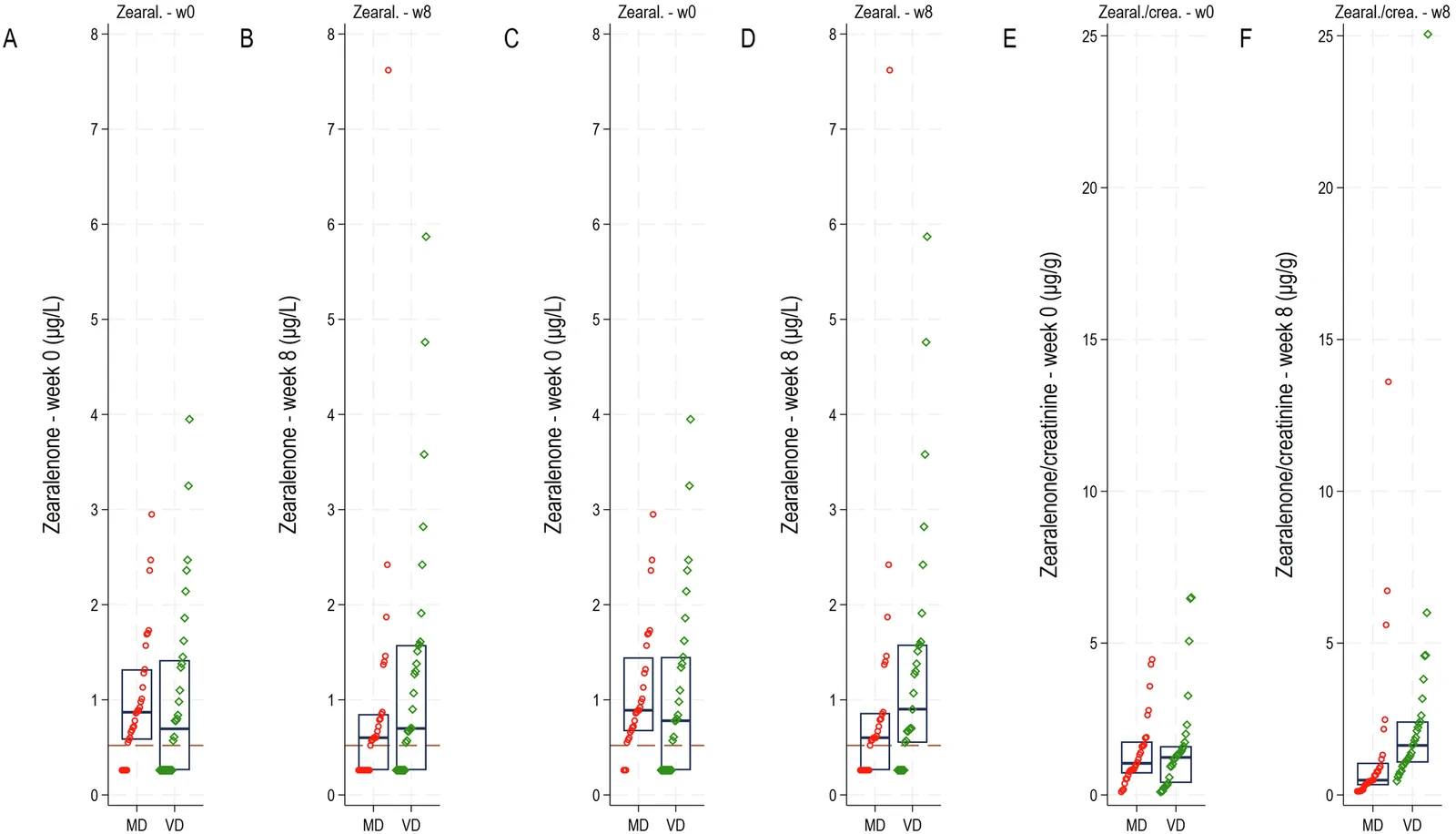
Strip plot displaying zearalenone excretion in urine at baseline (week 0) and after eight weeks into the study with a meat-rich diet (red color) or vegan diet (green color). **A**, **B** Both panels show the entire sample (including those observations below the detection limit) whereas panels **C**–**F** show only participants above the detection limit (0.52 μg/L). Panels **E**, **F** display creatinine-adjusted values (in μg/g). The horizontal dashed brown line marks the detection limit. MD Meat-Rich Diet, VD Vegan Diet. Based on *n* = 63 paired observations. Zearalenone concentrations were below the detection limit in *n* = 20 samples (31.75%) at baseline and *n* = 22 samples (34.92%) after eight weeks, respectively.

For additional insights, we visualized the results in a series of strip plots (Figs. 1–4 and Supplementary Fig. 3) in both the full sample and specifically restricted to those participants with urinary mycotoxin concentrations above the respective mycotoxin-specific detection level. The number of participants with mycotoxin concentrations below the detection limit is provided in the legend of each figure and in Supplementary Table 1. Diet- and time-specific numbers are provided in Supplementary Tables 2 and 3.

**Fig. 5:**
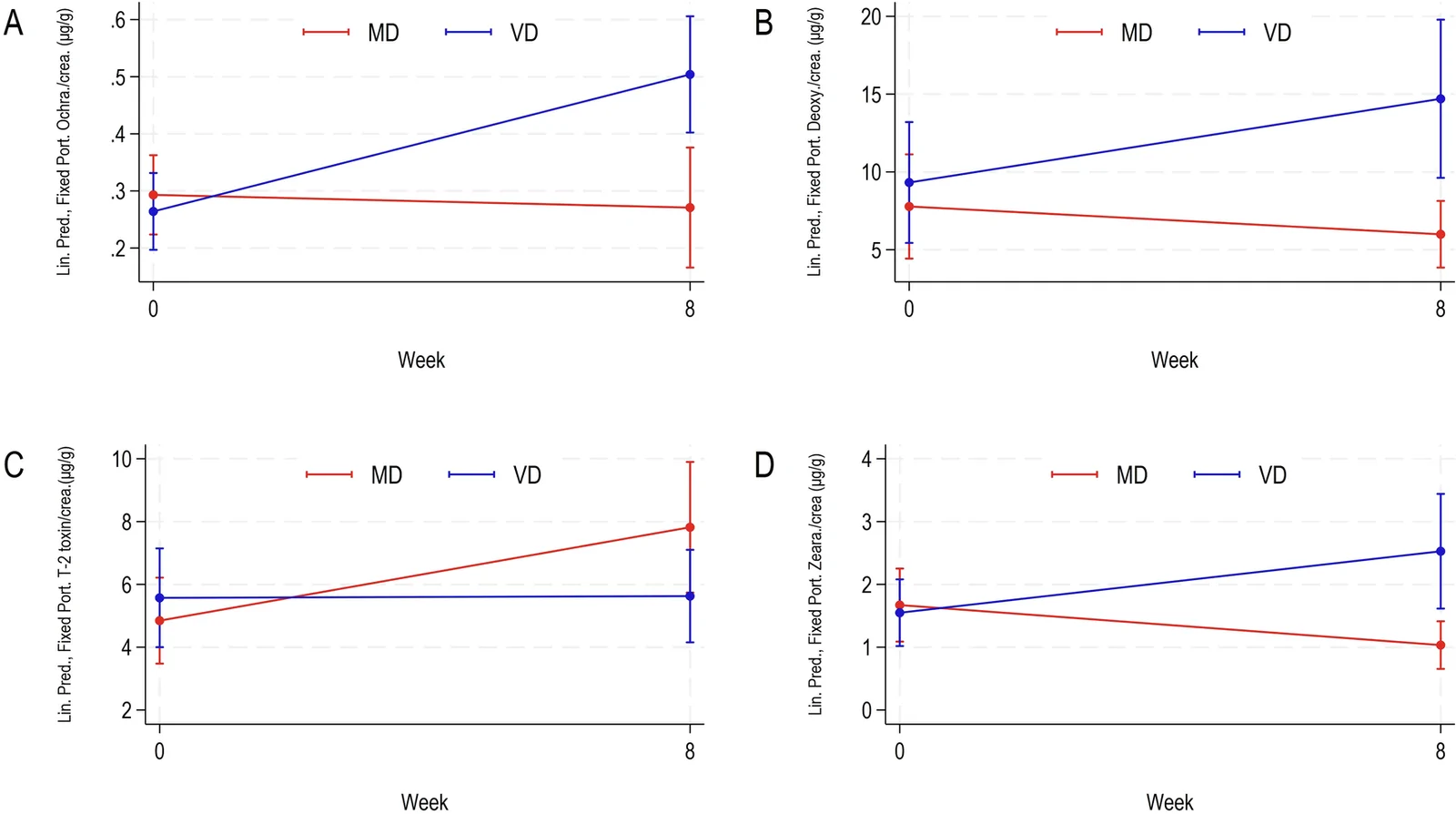
Mixed model repeated measures results: creatinine-adjusted mycotoxin excretion with the time-diet-interaction as a “fixed effect”. All models were fitted using restricted maximum likelihood and other modifications as discussed by Kenward and Roger^42,43^. Panels **A** (creatinine-adjusted ochratoxin A excretion in μg/g) and **B** (creatinine-adjusted deoxynivalenol excretion in μg/g) are based on *n* = 62 observations as one outlier was removed to highlight full cohort behavior over the casuality of one outlier and to ensure normal distribution of the residuals. To ensure normal distribution of the residuals in panels **B**, **C** (creatinine-adjusted T-2 toxin excretion in μg/g) and **D** (creatinine-adjusted zearalenone excretion in μg/g), we initially performed log-transformations. Despite the transformation, panel **C** had to be restricted to *n* = 61 observations to ensure normal distribution of the residuals. Instead of exponentiating the log-scale predictions to convert them back to concentrations in μg/g (which could result in biased predictions), we used an approach discussed by Huber and modified by Coveney^44,45^. All underlying models were statistically significant (*p* < 0.01) except for the one presented in **C** (*p* = 0.105). The upper limit of the reference range for the creatinine-adjusted mycotoxin values are as follows: deoxynivalenol: <4.85 μg/g; ochratoxin A < 0.40 μg/g; T-2 toxin <1.96 μg/g; zearalenone <0.83 μg/g.

Figure 5 displays mixed model repeated measures (MMRM) results. Panel A depicts creatinine-adjusted ochratoxin A excretion depending on diet at week 0 (baseline) and week 8. Stata’s contrast command suggested a significant diet-time-interaction term (*p* = 0.001). The predicted increase in the VD group in comparison to baseline (+0.24 (95%-CI: 0.13–0.35) μg/g) was statistically significant (*p* < 0.001) whereas no significant changes were observed in the MD group (-0.02 (95%-CI: −0.14–0.09) μg/g).

As for the creatinine-adjusted deoxynivalenol excretion in panel B, Stata’s contrast command likewise suggested a significant diet-time-interaction term (*p* = 0.029). Notably, predicted excretions differed at week 8 (5.99 (95%-CI: 3.85–8.13) *vs*. 14.70 (9.62–19.79) μg/g, *p* = 0.002). A significant increase in the VD group was observed (*p* = 0.046). Panel C depicts creatinine-adjusted T-2 toxin excretion. The diet-time-interaction term was not significant (*p* = 0.091) and neither was the between-group difference at week 8 (*p* = 0.092). Notably, the overall insignificant model (*p* = 0.105) suggested an increase in the creatinine-adjusted T-2 toxin excretion in the MD group (*p* = 0.019) aligning with Wilcoxon matched-pairs signed-rank test results.

In panel D, we present MMRM-based creatinine-adjusted zearalenone predictions. Stata’s contrast command suggested a significant diet-time-interaction term (*p* = 0.002). The difference between week 8 and baseline in the MD group was significant (*p* = 0.029) and the predicted between-group difference at week 8 was also significant (1.03 (95%-CI: 0.65–1.41) *vs*. 2.53 (95%-CI: 1.61–3.44), *p* = 0.003).

In a subsequent step, we sought to explore potential associations between the measured urinary mycotoxin concentrations and weighed food diary-based food group intake. Supplementary Table 4 displays daily food group intake in the full sample and in a diet-specific manner (MD vs. VD). No relevant differences in food groups were observed at baseline with one exception: average dairy food consumption was higher in those participants who were later assigned to the VD. Pronounced differences were found at week 8. In accordance with the study-specific instructions, self-reported meat, egg and dairy intake was zero in the VD group. Participants assigned to the MD had a median meat intake of two servings per day. Vegetable oil consumption was higher in the VD group and the same applied to nut, vegetable, legume and fruit intake. Plant yoghurts were almost exclusively consumed in the VD group whereas some participants in the MD group consumed non-dairy milk alternatives. No between-group differences were observed regarding whole and refined grain consumption.

Supplementary Tables 5 and 6 display Spearman’s rank-order correlations between food intake at week 8 of the dietary intervention and crude and creatinine-adjusted urinary mycotoxin concentrations, respectively. Moderate positive correlations were found between vegetable intake and all examined (creatinine-adjusted) mycotoxins (except for T-2 toxin). A positive correlation was also found for legume intake and deoxynivalenol excretion. Other noteworthy findings included a positive correlation between fish/seafood intake and T-2 toxin in the crude and creatinine-adjusted correlation, positive correlations between plant yoghurt intake and almost all mycotoxins (except for T-2 toxin) as well as a positive correlation between zearalenone excretion and whole grain intake. The results were subsequently visualized using scatter plots as many correlations were partly zero-inflated (e.g., zero servings of meat intake in the VD group). As such, we plotted spearman ranks to gain additional insights (Supplementary Fig. 4 + 5). The combination of both approaches suggested that true monotonic relationships may only exist for vegetable intake and deoxynivalenol excretion, whole grain intake and zearalenone excretion as well as vegetable intake and ochratoxin A excretion. For additional insights, we also ran restricted cubic spline regression with 3 knots to model more complex, non-linear relationships. Said models are visualized in Supplementary Fig. 6 and essentially confirmed potential associations between vegetable intake and creatinine-adjusted deoxynivalenol and ochratoxin A excretion. No convincing associations were found for all other food items. The isocaloric nature of the dietary intervention is displayed in Supplementary Fig. 7, highlighting that two diets with a similar energy level were compared for this analysis.

## Discussion

The present study used urinary samples obtained from an eight-weeks dietary intervention trial with two isocaloric arms (VD vs MD) to determine mycotoxin exposure following dietary modifications. Results suggested noticeable differences subsequent to the dietary intervention. MMRM results pointed at a significant diet-time interaction for ochratoxin A, with an increase in mycotoxin excretion in the VD group in comparison to baseline. Creatinine-adjusted deoxynivalenol excretion differed between both diet groups, with significantly higher values found in those assigned to the VD at week 8. MMRM results essentially pointed in the same direction. Creatinine-adjusted urinary zearalenone concentrations were likewise higher in those assigned to the VD at week 8. The only mycotoxin which increased in those assigned to the MD was T-2 toxin. Overall, our results suggested that diet may have a non-negligible impact on mycotoxin exposure. The food group analyses, however, are difficult to interpret because only few significant correlations were found and because each food group encompasses a wide array of food items. Regression models with cubic splines pointed at a higher deoxynivalenol and ochratoxin A excretion subsequent to a higher vegetable intake but must be viewed with caution due to the modest sample size. To the best of our knowledge, we present the first isocaloric vegan vs. omnivorous-diet based mycotoxin analysis in the context of a randomized-controlled trial. These findings warrant a thorough discussion in the context of the available literature on plant-based nutrition and mycotoxin exposure.

Higher mycotoxin levels in novel plant-based meat-, milk- and fish- analogues have recently attracted great attention^12,13^. However, there is also a study dating back to the early 2000s which has suggested that vegans could be exposed to a higher mycotoxin exposure with potentially detrimental health effects^20^. Other key studies in the field include the seminal work of Wells et al.^18^, Penczynski et al.^16^, Fleury et al.^19^ and Eriksen et al.^21^ and Halldorsson et al.^17^.

Wells et al. used food intake and urinary excretion data from *n* = 32 vegetarians and *n* = 31 omnivores in the United Kingdom^18^. The authors investigated deoxynivalenol excretion in a cross-sectional manner. Morning spot urine samples were obtained from all participants on two consecutive days. Food intake assessment strategies included semi-structured interviews and weighed food diaries, which were also used in our study. Deoxynivalenol was more frequently detected in urine samples from omnivores, which was not the case in our study (at week 8, deoxynivalenol was found in 48.39% of samples in the VD group and 31.25% of samples in the MD group, respectively). With the exception of males at day 2, total mean (crude) deoxynivalenol levels were higher in vegetarians in the UK sample. Aligning with our results, total creatinine-adjusted deoxynivalenol levels were on average lower in omnivores. No food group correlation analysis was reported in Wells et al.^18^.

The seminal work of Penczynski et al. from Germany was not restricted to a single mycotoxin^16^. In fact, Penczynski et al. examined 27 mycotoxins in both serum and urine in *n* = 72 individuals, thereof *n* = 36 vegans. Cross-sectional in design, the authors used fasting venous blood samples and 24-h urine samples. As such, our results are only partially comparable to theirs because we only determined mycotoxin excretion in urinary samples. While food consumption was also assessed with weighed food diaries, Penczynski et al. constructed *n* = 53 food groups which may allow for a more exact discrimination between food items. Eight different mycotoxins were detected in vegans, whereas only six distinct mycotoxins were detected in omnivores. Urinary mycotoxin patters differed significantly between both groups (*p* = 0.030). Serum ochratoxin A levels were twofold higher in vegans than in omnivores (median 0.24 ng/mL vs. 0.12 ng/mL; *p* < 0.001). Ochratoxin levels were associated with the intake of “vegan products” (*r* = 0.50, *p* < 0.001) as well as “pasta & rice” (*r* = 0.33, *p* = 0.006) although sensitivity analyses suggested to warrant a cautious interpretation. Both food intake associations could not be replicated in our study, however, we also found significantly higher ochratoxin A levels in vegans (though in urine and not in the serum of participants).

Another major analysis in the field stems from France and was conducted by Fleury et al. based on the NutriNet Santé study^19^. The sample was much larger than in Wells et al. and Penczynski et al. and included *n* = 1766 French vegetarians (including 188 vegan individuals) aged 18 to 81 years^19^. Like in our study, aflatoxin, deoxynivalenol, fumonisin, ochratoxin A, zearalenone and T-2 toxin were assessed. This cross-sectional study relied on web-based dietary records on three non-consecutive days over a two weeks period. T-2 toxin was higher in vegetarians in comparison to the general French population. This finding is contrary to ours, as the MMRM in our study suggested an increase with the MD and stable urinary values when adopting a VD for eight weeks. “Pasta” (with 32.5%), “bread and dried breads” (26.9%) and “rice and wheat products” were considered the main contributors (14.2%) in the French sample. Notably, such associations could not be replicated in our study. Future research is thus warranted.

Finally, two studies from the Nordic countries warrant discussion. Eriksen et al. investigated the fusarium mycotoxin deoxynivalenol in a Norwegian sample of *n* = 257 individuals, thereof *n* = 30 vegetarians^21^. Deoxynivalenol and its metabolites were detected in almost all study participants (*n* = 256) using morning spot urine samples. Food intake was assessed using an open food diary on two days. The only person in whom deoxynivalenol could not be measured did not consume any grain products. Values in vegetarians were equal to those of the general population^21^.

Very recently, Halldorsson et al. published results from *n* = 218 individuals from Iceland (thereof *n* = 47 vegans)^17^. Participants’ diet was assessed using two 24-h recalls. The authors also used spot urine to quantify T-2 toxin, deoxynivalenol, ochratoxin A, and zearalenone. This study is noteworthy because it did not confirm the hypothesis that vegans are exposed to higher mycotoxin levels. In fact, deoxynivalenol and ochratoxin were detected in all omnivorous samples but in 99% and only 77% of vegan samples, respectively. Creatinine-adjusted urinary concentrations of deoxynivalenol were similar among omnivores and vegans (median: 2.04 vs. 1.84 ng/g creatinine), while ochratoxin concentrations were 10-fold lower in vegans than in omnivores (*p* < 0.001). Again, this study points in an opposite direction when compared to our results^17^.

While other noteworthy studies exist (e.g., modeling studies by Ramos et al.^24^), we deemed it important so synthesize the results from Wells et al., Penczynski et al., Fleury et al., Eriksen et al. and Halldorsson et al. because said studies are the major studies in the field that recruited either vegetarians or vegans^16–19,21^. A comparative analysis of these studies suggests that results are often inconsistent, although most studies pointed at higher deoxynivalenol levels in those reporting plant-based diets (except the work from Halldorsson et al.^17^). The fact that previous food group analyses are not consistent suggests either a high intra-individual variation in risk profiles or very specific food items that contribute to the mycotoxin burden in vegetarians and vegans.

In recent years, increasing attention has been paid to novel food items such as plant-based milk and meat analogues^12,13^, which mimic their animal-sourced counterparts. While there are now numerous reports that suggest that such foods could be important contributors, their overall impact remains to be determined. The fact that a study from the early 2000s has already reported increased mycotoxin levels in vegans does not support the hypothesis that such foods are the main mycotoxin source^20^. In fact, most of these foods were unavailable back in these days (e.g., ultra-processed plant-based meat analogues). Nevertheless, it must be acknowledged that contaminated grains are likely the main source of mycotoxins in the human diet. This applies for example to T-2 toxin^25^. Against this background, we cannot explain the higher T2 values in those assigned to the MD group in our study.

The higher ochratoxin A levels in our study in those assigned to the VD are biologically plausible; ochratoxin A is frequently found in foods of plant origin (such as cereal products, coffee, vegetable, rice, raisins, wine)^26^. The results are directionally consistent with a recently published multi-mycotoxin biomonitoring study in Italian adults, that demonstrated higher exposure to mycotoxins among individuals with an increased consumption of plant-based foods^27^. Especially vegetables were consumed twice as frequent in VD participants as compared to MD participants in our study. Likewise, higher deoxynivalenol levels in VD participants are also biologically plausible, as this mycotoxin is frequently found in maize-based products^28^, breads and rolls^28^, as well as wheat and wheat flour products^29,30^.

As for the plant-based milk alternatives, it remains important to emphasize that intakes at the end of the study did not differ between VD and MD participants. Participants were free to select the food items they wanted (as long as their selections were in accordance with the study-specific criteria). Plant-based milks are by themselves a large and inhomogeneous group and a more distinct investigation (e.g., associations with single food items such as oat or soy milk alone) might be warranted in future studies.

This study has several additional limitations worth consideration. Although weighed food diaries were used, dietary intake data is always subject to recall and reporting bias. The former may be minimized with weighed food diaries whereas the latter may not be ruled out. In a few instances (*n* = 6), participants provided food diaries for only two days. In such cases, the average food group intake estimation was not based on three days but on two days only. Spices, which may also be an important source of mycotoxins, were not carefully documented by many participants. While the food category assignment in the analysis was based on established procedures and the extant literature (see below), a reservation must be made that the underlying food groups were relatively broad and encompassed foods with varying mycotoxin contamination profiles. This could potentially explain the lack of relevant food-specific associations that were expected based on the extant literature. Beyond that, participants were instructed to complete the weighed food diaries as close as possible to their following appointment at the study center (see Supplementary Fig. 1). Appointments were usually scheduled on Mondays. Participants were instructed to complete weighed food diaries on two weekdays and one weekend day. Thus, the weighed food diaries do not necessarily cover all preceding days of the biosampling (either the preceding Saturday or Sunday was not covered). Nevertheless, all food diaries were collected in close proximity to the biosampling of spot urine. Mycotoxins were neither measured in 24-h urine samples nor in serum samples. Instead, morning urine spot samples were used. The underlying limitations of this approach have been discussed in detail earlier^16^. Finally, no corrections for multiple testing (e.g., the correlations) was made, increasing the chance for a type I error.

As for the strengths, we report data from a randomized-controlled trial with pre vs. post dietary intervention comparisons and between-group comparisons. The employed weighed food diaries are most valuable for the assessment of highly specific dietary choices as often found in vegans^16^. An additional strength is the dual presentation of crude and creatinine-adjusted mycotoxin urine levels, which appears particularly critical when comparing vegans to omnivores (see Supplementary Fig. 8)^31^. Abraham et al. argued that researchers should keep in mind that creatinine-adjustments of biomarkers measured in spot urine could lead to falsely lower results in omnivores^31^. A main reason for this is the absent consumption of meat and fish as exogenous sources of creatinine in vegans^31^. Abraham et al. also argued that the averagely lower muscle/fat-free mass of vegans (as shown by Burke et al. and Bartholomae et al.^32,33^) could result in differences in the creatinine excretion of vegans and omnivores^31^. Notably, we did not examine long-term vegans in this study and the intervention was restricted to eight weeks rendering large dietary-induced fat free mass differences unlikely. To account for the aforementioned creatinine excretion differences, Abraham et al. recommended the usage of 24-h urine samples or, if not available, the usage of specific gravity adjustments for biomarker measurements in spot urine^31^. We acknowledge that both were not available for the present study. Controversies persists around vegan vs. omnivore urinary profiles comparisons and future research is required to confirm these hypotheses. In the meantime, it remains advisable to report both crude (absolute) and creatinine-adjusted (normalized) values^34^. The latter remains the widely considered gold standard^35^. Finally, we deem it important to emphasize that the herein presented upper limits of the reference ranges for the creatinine-adjusted mycotoxin values (as provided by the contract laboratory) do not reflect safety thresholds. The present study can only point at differences in diet-dependent exposure patterns, direct health outcomes were not included.

Additional well designed and sufficiently powered randomized studies are required to determine the exposure of vegans to mycotoxins. Substantial efforts from a public health and food safety perspective including strict regulations and food quality controls are deemed necessary in general to reduce the level of mycotoxin exposure for all humans regardless of their diet.

Results from the present study pointed at different mycotoxin exposure patterns for short-term vegans and omnivores subsequent to an isocaloric dietary intervention. Mycotoxin exposure in humans thus requires attention from a food safety, public health and toxicology perspective. Whether the apparently higher exposure in participants assigned to the vegan group suggested by the present study has potentially detrimental health effects remains to be investigated in future studies. This is important, because a regular high intake of plant-sourced foods, including fruits, vegetables and legumes, as found in whole food vegan diets, has been associated with numerous health benefits^36,37^. The herein presented results are not meant to discourage individuals to adopt such a diet. To the contrary, we encourage plant-forward diets due to their known health benefits and call for increased attention to adopt effective means to reduce mycotoxin exposure for all humans.

## Methods

### Study population and design

This trial was prospectively registered in a Clinical Trial Register (DRKS00031541) on 27 March 2023. The study was performed in accordance with the Declaration of Helsinki. The ethical committee of the University Medical Center of Freiburg approved this study (approval number: 22-1474-S1; 01/2023) before onset. Written and oral informed consent was obtained from all study participants. Mycotoxin measurements in urine were covered by the following amendment: SS-1474_1-S1 (12/2025). The study population and design have been described earlier in great detail^22,23^. In brief, urine samples were taken from an eight-weeks dietary intervention study which compared the effects of an isocaloric vegan diet (VD) and a meat-rich diet (MD) on immune-related factors. All study participants were omnivores upon recruitment and then randomly assigned to either a VD (which did not permit any animal-based foods for eight weeks) or a MD (which included the consumption of at least 150 g of meat on a daily basis). No restrictions were imposed regarding the type of meat, although fish and fish products were not considered to fulfill the study criteria. Participants in the VD group were encouraged to select whole foods; this focus was not set in the MD group. Participants were free to select the foods they liked as long as their choices were in accordance with the requirements of the study protocol. No meals were provided. Study participants in both groups received multiple group seminars, nutritional coaching sessions, and nutrient-specific weekly newsletters to increase dietary adherence.

Major recruitment-related inclusion criteria and exclusion criteria have been discussed earlier^22,23^. In brief, healthy individuals aged 18 to 60 years with a BMI ranging from 21 to 30 kg/m^2^ were considered for the study. Exclusion criteria were as follows: chronic health conditions, pregnancy, lactation, clinically-relevant allergies, eating disorders as well as alcohol (>20 g/d) and daily nicotine intake. Interested individuals who reported a plant-based diet prior to their study participation were excluded, as well.

Prior to the random group assignment, all participants underwent a one-week run-in phase which included a mixed diet as per the dietary recommendations of the The German Nutrition Society (DGE). Briefly, these recommendations include a daily consumption of dairy products, at least five servings of fruits and vegetables per day, fish twice a week and a maximum of 300 g of meats per week. Urine samples were collected at baseline upon completion of the run-in-phase and after eight weeks. Urine samples were collected in the early morning hours; spot urine samples were collected (24-h morning urine samples were not available in this study). Supplementary Fig. 1 displays a study scheme that displays the most important time points during the study. All participants were asked to take part in nutrition education sessions and group meetings for diet-specific nutritional instructions.

### Mycotoxin assessment

Mycotoxins were measured in morning spot urine at baseline (after a one-week mixed diet) and after 8 weeks of a VD or MD, respectively. To detect and quantify the presence of mycotoxins in urine samples, a modified SAFIA mycotoxin screening assay (SAFIA, SAFIA Technologies GmbH, Berlin, Germany) was performed and run by IMD Institut für Medizinische Diagnostik Berlin‑Potsdam GbR, Nicolaistraße 22, 12247 Berlin‑Steglitz, Deutschland. SAFIA is a particle-based multiplex immunoassay, which enables the precise and efficient simultaneous analysis of multiple analytes in one approach. The SAFIA test uses unique fluorescent color-coded microparticles. Each code corresponds to a specific analyte, allowing simultaneous detection of the different mycotoxins in one sample. The assay is based on a competitive immunoassay principle. Analyte molecules (mycotoxins) are covalently bound to microparticles, serving as antigens for the primary antibodies. These antibodies binds either to the particle-bound or the free mycotoxins in the urine samples. One primary antibody is used for each single mycotoxin. The primary antibodies are detected by secondary fluorescence antibodies. Flow cytometry measurements detect both, the color code of each single particle and the fluorescence signal from the secondary antibodies. Fluorescence intensity measured on each uniquely coded particle can be directly linked to the concentration of the corresponding mycotoxin. The following six mycotoxins with the indicated detection limit (d.l.) can be analyzed: aflatoxin (d.l. 0.21 µg/L), deoxynivalenol (d.l. 2.79 µg/L), fumonisin (d.l. 3.55 µg/L), ochratoxin A (d.l. 0.11 µg/L), T-2 toxin (d.l. 1.46 µg/L), zearalenone (d.l. 0.52 µg/L). Control particles, artificial urine blanks, spiked standards, and a fixation step to prevent antibody crosslinking ensure assay accuracy, compensate matrix effects and confirm sensitivity in every run. Mycotoxin concentrations are displayed in their crude form and with creatinine-adjusted values (in μg/L and in μg/g creatinine, respectively). The upper limit of the reference range for the creatinine-adjusted mycotoxin values provided by the contract laboratory were as follows: aflatoxin: <0.28 μg/g; deoxynivalenol: <4.85 μg/g; fumonisin: <5.43 μg/g; ochratoxin A: <0.40 μg/g; T-2 toxin: <1.96 μg/g; zearalenone: <0.83 μg/g.

### Dietary intake assessment

Weighed food diaries were used to capture participants’ dietary intake (Supplementary Fig. 1). Participants completed weighed food diaries on three different days during week 1 (upon following a mixed, balanced diet) and on three different days during week 9 of the study (upon following a VD or MD). The procedure has been described elsewhere in great detail^22,23^. For the weighed food diaries, we instructed participants to weigh all solid foods and beverages to the nearest 0.1 g or ml, respectively. When required, participants were provided a calibrated precision scale. The time and place of consumption, the exact product name including nutrient-relevant packaging details, the weighed quantity and remaining quantity were queried. The completeness of all products was verified using semi-structured interviews. Food-derived nutrients were analyzed using NutriGuide® dietetic software (version 4.9, Nutri-Science GmbH, Jacobistr. 39, 79104 Freiburg im Breisgau, Germany). Beyond that, we calculated the reported daily number of servings for 21 distinct food groups. The latter included whole grains, fruits, vegetables, nuts, legumes, vegetable oils, tea/coffee, fruit juices, refined grains, potatoes, sugar-sweetened beverages, sweet desserts, dairy, animal fat, fish and seafood, eggs, meat and several food groups specific to plant-based diets (e.g., plant-based milk alternatives). The full list including serving sizes is displayed in Supplementary Table 4 and was based on the extant literature^38^.

### Statistical analysis

The statistical analysis was performed by PSK and MAS using Stata (StataCorp. 2025. Stata Statistical Software: Release 19. College Station, TX: StataCorp LLC). Data distribution was evaluated using the Shapiro-Wilk test in Stata and visually with histograms. For normally distributed variables, we provided means and standard deviations. For variables which were not normally distributed, we provided medians and interquartile ranges in parenthesis. Correlation analyses including Pearson product-moment correlations and Spearman rank-order correlations were run to examine potential associations between urinary mycotoxin concentrations and food groups and clinical parameters.

The urinary mycotoxin analysis included various measurements below the aforementioned lower detection limits. The missing values were not simply ignored but handled based on the recommendations of Giskeødegård and Lydersen^39^. Against the background that simulation studies have shown that imputation with zero is “*generally not advisable*”, we followed Giskeødegård and Lydersen’s advice and went for an imputation with half the detection limit because it is the method that introduces the least bias into the estimates^39,40^.

Depending on the data distribution, non-parametric tests (Wilcoxon rank-sum test, Mann–Whitney-U-Test) and parametric tests (paired and unpaired two-tailed t-tests) were used to examine statistically significant within-group (*pre* vs. *post*-intervention) and between-group differences (*at baseline and the end of the study*) in continuous variables. Scatter plots, strip plots and margins plots were used to visualize the data^41^.

Finally, we used MMRM (mixed model repeated measures) which were deemed adequate for the study context. Said models are a popular approach in the context of randomized clinical trials, which repeatedly measure individuals over time (in our case two measurements)^42^. Following an approach by Bartlett, we specified no participant level random effect^42^. Instead, our model modeled the correlation with the repeated measures over time by specifying that the residual errors were correlated. This was done to reduce the odds of model misspecification. We assumed residual errors to be from an unstructured covariance matrix. As for the “fixed effects”, we specified the time-diet-interaction. The model was fitted using restricted maximum likelihood and other modifications as discussed by Kenward and Roger^42,43^. Stata’s contrast command with the diet-time-interaction term was then used as a joint test of the interaction including main effects. To ensure normal distribution of the residuals in some models, we initially performed log-transformations. Instead of exponentiating the predictions to convert them back to concentrations in μg/g (which could result in biased predictions), we then followed an approach discussed by Huber and modified by Coveney^44,45^. Statistical significance was determined at *α* = 0.05. No corrections for multiple testing were made.

## Supplementary information

Supplementary Information

## Data Availability

The datasets generated and/or analyzed during the current study are not publicly available due to institutional ethics restrictions but are available from the corresponding author on reasonable request.
