# Supplementary material for: Mycotoxin exposure following an eight-week vegan or meat-rich dietary intervention: a randomized-controlled trial
Source: NPJ Sci Food. 2026 Sep 18;10:284. doi: 10.1038/s41538-026-01152-4 (PMC13594211; doi:10.1038/s41538-026-01152-4)

Supplementary Data: Mycotoxin exposure following an eight-week vegan or meat-rich dietary intervention: a randomized-controlled trial

Supplementary Tables

Supplementary Table 1

Supplementary Table 1: Number of samples below the detection limit in the full sample (*n* = 63) at baseline and at week 8 of the study, respectively.

Mycotoxin	Number of samples below the detection limit (*n* = 63)	
	Week 0	Week 8
Aflatoxin	*n* = 55 (87.3%)	*n* = 57 (90.48%)
Deoxynivalenol	*n* = 28 (44.44%)	*n* = 25 (39.68%)
T-2 toxin	*n* = 20 (31.75%)	*n* = 17 (26.98%)
Ochratoxin A	*n* = 21 (33.33%)	*n* = 12 (19.05%)
Zearalenone	*n* = 20 (31.75%)	*n* = 22 (34.92%)
Fumonisin	*n* = 63 (100%)	*n* = 61 (96.83%)

Supplementary Table 2

Supplementary Table 2: Number of samples below the detection limit at baseline depending on diet group (vegan diet (VD) *vs*. meat-rich diet (MD)).

Mycotoxin	Number of samples below the detection limit at week 0 (*n* = 63)	
	VD (*n* = 32)	MD (*n* = 31)
Aflatoxin	*n* = 30 (93.75%)	*n* = 25 (80.65%)
Deoxynivalenol	*n* = 14 (43.75%)	*n* = 14 (45.16%)
T-2 toxin	*n* = 10 (31.25%)	*n* = 10 (32.26%)
Ochratoxin A	*n* = 14 (43.75%)	*n* = 7 (22.58%)
Zearalenone	*n* = 14 (43.75%)	*n* = 6 (19.35%)
Fumonisin	*n* = 32 (100%)	*n* = 31 (100%)

Supplementary Table 3

Supplementary Table 3: Number of samples below the detection limit at week 8 depending on diet group (vegan diet (VD) *vs*. meat-rich diet (MD)).

Mycotoxin	Number of samples below the detection limit at week 8 (*n* = 63)	
	VD (*n* = 32)	MD (*n* = 31)
Aflatoxin	*n* = 27 (84.38%)	*n* = 30 (96.77%)
Deoxynivalenol	*n* = 10 (31.25%)	*n* = 15 (48.39%)
T-2 toxin	*n* = 14 (43.75%)	*n* = 3 (9.68%)
Ochratoxin A	*n* = 6 (18.75%)	*n* = 6 (19.35%)
Zearalenone	*n* = 10 (31.25%)	*n* = 12 (38.71%)
Fumonisin	*n* = 31 (96.88%)	*n* = 30 (96.77%)

Supplementary Table 4

Supplementary Table 4: Daily food group servings consumed at baseline (week 0) and after eight weeks of the dietary intervention

**Food Group (number of servings *(n)* per day)**	**Week 0**				**Week 8**			
	Full Sample (n = 63)	VD group (n= 32)	MD group (n = 31)	*p*-value	Full Sample (n = 63)	VD group (n= 32)	MD group (n = 31)	*p*-value
**Whole grains** *(n)*	1.41 (1.93)	1.40 (1.66)	1.61 (2.47)	0.921[a]	1.36 (1.69)	1.56 (1.86)	1.18 (1.50)	0.441[a]
**Fruits** *(n)*	0.62 (0.71)	0.59 (0.53)	0.75 ± 0.50	0.751[a]	0.69 (0.94)	1.00 (0.71)	0.47 (0.69)	**0.002**[a]
**Vegetables** *(n)*	1.57 (1.32)	1.57 (1.38)	1.58 (1.39)	0.759[a]	1.62 (1.65)	2.10 (1.38)	1.24 (1.82)	**0.013**[a]
**Nuts** *(n)*	0.20 (1.03)	0.33 (0.80)	0.13 (1.07)	0.445[a]	0.53 (1.33)	0.99 (1.14)	0.00 (0.63)	**<0.001**[a]
**Legumes** *(n)*	0.10 (0.60)	0.11 (0.62)	0.08 (0.49)	0.734[a]	0.37 (1.00)	0.91 (1.00)	0.20 (0.37)	**<0.001**[a]
**Vegetable oils** *(n)*	0.67 (1.33)	0.67 (0.96)	0.68 (1.53)	0.702[a]	1.10 (1.85)	1.50 (2.06)	0.80 (1.50)	**0.003**[a]
**Tea/coffee** *(n)*	0.20 (1.33)	0.60 (1.33)	0.00 (0.68)	0.215[a]	0.20 (0.78)	0.23 (0.87)	0.20 (0.71)	0.875[a]
**Fruit juices** *(n)*	0.00 (0.04)	0.00 (0.00)	0.00 (0.08)	0.372[a]	0.00 (0.01)	0.00 (0.01)	0.00 (0.03)	0.890[a]
**Refined grains** *(n)*	3.68 ± 1.92	3.57 ± 2.15	3.78 ± 1.69	0.670[b]	3.00 (3.36)	3.04 (3.33)	2.90 (3.63)	0.973[a]
**Potatoes** *(n)*	0.35 (0.62)	0.35 (0.63)	0.33 (0.42)	0.434[a]	0.10 (0.44)	0.13 (0.48)	0.07 (0.42)	0.871[a]
**Sugar-sweetened beverages** *(n)*	0.00 (0.36)	0.00 (0.22)	0.00 (0.36)	0.762[a]	0.00 (0.44)	0.00 (0.44)	0.00 (0.53)	0.343[a]
**Sweet dessert** *(n)*	0.73 (1.58)	0.82 (1.54)	0.72 (1.69)	0.624[a]	0.67 (1.10)	0.67 (1.04)	0.75 (1.24)	0.735[a]
**Animal fat** *(n)*	0.00 (0.67)	0.13 (0.97)	0.00 (0.57)	0.189[a]	0.00 (0.00)	0.00 (0.00)	0.00 (0.90)	**<0.001**[a]
**Dairy** *(n)*	2.93 (2.31)	3.70 ± 2.15	2.68 ± 1.51	**0.035**[b]	0.00 (2.64)	0.00 (0.00)	2.64 (2.99)	**<0.001**[a]
**Eggs** *(n)*	0.02 (0.67)	0.10 (0.67)	0.00 (0.83)	0.613[a]	0.00 (0.67)	0.00 (0.00)	0.67 (1.00)	**<0.001**[a]
**Fish/seafood** *(n)*	0.00 (0.25)	0.00 (0.08)	0.00 (0.42)	0.674[a]	0.00 (0.00)	0.00 (0.00)	0.00 (0.13)	**0.004**[a]
**Meat** *(n)*	0.80 (1.15)	0.92 (0.93)	0.59 (1.37)	0.079[a]	0.00 (2.04)	0.00 (0.00)	2.04 (2.33)	**<0.001**[a]

**Misc. animal-based foods** *(n)*	0.00 (0.47)	0.00 (0.48)	0.00 (0.33)	0.611 [a]	0.00 (0.00)	0.00 (0.00)	0.00 (0.92)	**<0.001** [a]
**Plant milk** *(n)*	0.10 (0.56)	0.00 (0.52)	0.28 (0.63)	0.168 [a]	0.33 (0.72)	0.39 (0.98)	0.13 (0.67)	0.238 [a]
**Plant yoghurt** *(n)*	0.00 (0.00)	0.00 (0.00)	0.00 (0.00)	1.000 [a]	0.00 (0.13)	0.11 (0.59)	0.00 (0.00)	**<0.001** [a]
**Misc. plant-based foods** *(n)*	0.32 (1.00)	0.17 (1.28)	0.50 (0.86)	0.913 [a]	0.82 (2.46)	2.20 (2.41)	0.23 (0.72)	**<0.001** [a]

Supplementary Table 4 legend: Serving sizes for the investigated food groups were as follows: *"whole grains"*: pasta & rice: 85g, breakfast cereal & bread: 50g; *"fruit"*: fresh fruit: 250g, dried fruit: 25g; *"vegetables"*: fresh vegetables: 200g, salad: 100g, dried vegetables: 25g*; "nuts":* 25g*; "legumes*": 125g; *"vegetable oil":* 10ml/10g; *"tea/coffee*": 250ml; *"fruit juice*s": 200ml; *"refined grains":* pasta & rice: 85g, breakfast cereal & bread: 50g; *"potatoes":* fresh: 200g, dried/chips: 40g; *"sugar-sweetened beverages"*: 250 ml; "*sweet dessert":* chocolate and cookies: 50g, jelly sweets: 25g, jam & Nutella: 30g, honey, maple sirup & agave syrup: 10g, sorbet: 60g; *"animal fat"*: 10ml/10g; *"dairy"*: yoghurt, milk, buttermilk & quark: 200g, cream: 50g, cheese and cream-cheese: 30g, dairy-based-protein-powder: 30g, ice cream: 60g; *"eggs":* 1 egg*;* "fish and seafood": 100g; *"meat*": white & red meat: 125g, sausage and smoked meat: 20g; *"misc. animal based foods*": mayonnaise or other creamy salad dressings: 30g; *"plant based milk*": 200ml; *"plant based yoghurt*": 200g; *"misc. plant based food*": vegan meat & dairy alternatives: serving sizes of non-vegan counterparts were used, spreads & sauces: 30g. Normally distributed data displayed as mean ± standard deviation, non-normally distributed data displayed as median (interquartile range). [a] = based on Mann-Whitney-U rank sum test. [b]=based on t-test. Misc. = miscellaneous. MD = Meat-Rich Diet; VD = Vegan Diet. *P*-values <0.05 are shown in bold.

Supplementary Table 5

Supplementary Table 5: Spearman's rank-order correlation between crude urinary mycotoxin concentrations and food groups intake at week 8 of the dietary intervention.

**Food Group (number of servings *(n)*)**	**Week 8: Crude urinary mycotoxin concentrations (µg/L)**			
	**Deoxynivalenol**	**T-2 toxin**	**Ochratoxin A**	**Zearalenone**
**Whole grains** *(n)*	ρ: 0.129<br>*p*-value: 0.312	ρ: -0.127<br>*p*-value: 0.320	ρ: 0.054<br>*p*-value: 0.676	ρ: 0.188<br>*p*-value: 0.140
**Fruits** *(n)*	ρ: 0.117<br>*p*-value: 0.362	ρ: -0.198<br>*p*-value: 0.121	ρ: 0.070<br>*p*-value: 0.584	ρ: 0.229<br>*p*-value: 0.071
**Vegetables** *(n)*	ρ: 0.184<br>*p*-value: 0.149	**ρ: -0.361**<br>***p*-value: 0.004**	ρ: 0.018<br>*p*-value: 0.891	ρ: -0.044<br>*p*-value: 0.773
**Nuts** *(n)*	ρ: -0.039<br>*p*-value: 0.761	ρ: -0.130<br>*p*-value: 0.308	ρ: -0.132<br>*p*-value: 0.300	ρ: -0.035<br>*p*-value: 0.786
**Legumes** *(n)*	ρ: 0.009<br>*p*-value: 0.947	ρ: 0.226<br>*p*-value: 0.075	ρ: -0.097<br>*p*-value: 0.447	ρ: -0.036<br>*p*-value: 0.781
**Vegetable oils** *(n)*	ρ: 0.085<br>*p*-value: 0.507	ρ: -0.173<br>*p*-value: 0.174	ρ: -0.023<br>*p*-value: 0.856	ρ: 0.015<br>*p*-value: 0.904
**Tea/coffee** *(n)*	ρ: 0.023<br>*p*-value: 0.860	ρ: -0.037<br>*p*-value: 0.775	ρ: -0.047<br>*p*-value: 0.717	ρ: -0.043<br>*p*-value: 0.737
**Fruit juices** *(n)*	ρ: 0.029<br>*p*-value: 0.818	ρ: 0.092<br>*p*-value: 0.477	ρ: 0.073<br>*p*-value: 0.570	ρ: -0.019<br>*p*-value: 0.886
**Refined grains** *(n)*	ρ: 0.055<br>*p*-value: 0.668	ρ: 0.081<br>p-value: 0.529	ρ: 0.070<br>*p*-value: 0.584	ρ: 0.008<br>*p*-value: 0.951
**Potatoes** *(n)*	ρ: 0.091<br>*p*-value: 0.476	ρ:-0.087<br>*p*-value: 0.498	ρ: 0.134<br>*p*-value: 0.294	ρ: 0.058<br>*p*-value: 0.652
**SSB** *(n)*	ρ: -0.026<br>*p*-value: 0.845	ρ: 0.010<br>*p*-value: 0.938	ρ: -0.034<br>*p*-value: 0.790	ρ: 0.070<br>*p*-value: 0.584
**Sweet dessert** *(n)*	ρ: 0.109<br>*p*-value: 0.395	ρ: 0.072<br>*p*-value: 0.575	ρ: -0.160<br>*p*-value: 0.210	ρ: 0.010<br>*p*-value: 0.938
**Animal fat** *(n)*	ρ: -0.063<br>*p*-value: 0.629	ρ: 0.161<br>*p*-value: 0.210	ρ: -0.005<br>*p*-value: 0.969	ρ: -0.137<br>*p*-value: 0.285
**Dairy** *(n)*	ρ: -0.143<br>*p*-value: 0.262	**ρ: 0.279**<br>***p*-value: 0.027**	ρ: -0.055<br>*p*-value: 0.667	ρ: -0.209<br>*p*-value: 0.100
**Egg** *(n)*	ρ: -0.180<br>*p*-value: 0.156	ρ: -0.064<br>*p*-value: 0.618	ρ: -0.064<br>*p*-value: 0.618	ρ: -0.182<br>*p*-value: 0.153
**Fish/seafood** *(n)*	ρ: -0.122<br>*p*-value: 0.346	**ρ: 0.322**<br>***p*-value: 0.010**	ρ: -0.125<br>*p*-value: 0.331	ρ: -0.069<br>*p*-value: 0.599
**Meat** *(n)*	ρ: -0.182<br>*p*-value: 0.153	**ρ: 0.334**<br>***p*-value: 0.008**	ρ: -0.073<br>*p*-value: 0.567	ρ: -0.196<br>*p*-value: 0.124
**Misc. animal-based foods** *(n)*	ρ: -0.151<br>*p*-value: 0.237	ρ: 0.197<br>*p*-value: 0.123	ρ: 0.186<br>*p*-value: 0.144	ρ: 0.098<br>*p*-value: 0.443
**Plant milk** *(n)*	ρ: 0.085<br>*p*-value: 0.507	ρ: 0.049<br>*p*-value: 0.705	ρ: 0.090<br>*p*-value: 0.480	ρ: 0.229<br>*p*-value: 0.072
**Plant yoghurt** *(n)*	ρ: 0.171<br>*p*-value: 0.181	**ρ: -0.264**<br>***p*-value: 0.036**	ρ: 0.005<br>*p*-value: 0.971	ρ: 0.118<br>*p*-value: 0.355

Misc. plant-based foods *(n)*	ρ: 0.033<br>*p*-value: 0.796	ρ: -0.214<br>*p*-value: 0.092	ρ: -0.007<br>*p*-value: 0.959	ρ: -0.011<br>*p*-value: 0.929

Supplementary Table 5 legend: SSB = Sugar-Sweetened Beverages. Misc- = Miscellaneous. Spearman's rank-order correlations based on observations of *n* = 63 participants. *P*-values <0.05 are shown in bold.

Supplementary Table 6

Supplementary Table 6: Spearman's rank-order correlation between creatinine-adjusted urinary mycotoxin concentrations and food groups intake at week 8 of the dietary intervention.

**Food Group (number of servings *(n)*)**	**Week 8: Creatinine-adjusted urinary mycotoxin concentrations (µg/g)**			
	**Deoxynivalenol**	**T-2 toxin**	**Ochratoxin A**	**Zearalenone**
**Whole grains** *(n)*	ρ: 0.243<br>*p*-value: 0.055	ρ: 0.072<br>*p*-value: 0.575	**ρ: 0.274<br>*p*-value: 0.030**	**ρ: 0.330<br>*p*-value: 0.009**
**Fruits** *(n)*	ρ: 0.202<br>*p*-value: 0.113	ρ: -0.187<br>*p*-value: 0.14	ρ: 0.223<br>*p*-value: 0.080	**ρ: 0.291<br>*p*-value: 0.021**
**Vegetables** *(n)*	**ρ: 0.483<br>*p*-value: <0.001**	ρ: -0.064<br>*p*-value: 0.617	**ρ: 0.442<br>*p*-value: <0.001**	**ρ: 0.340<br>*p*-value: 0.007**
**Nuts** *(n)*	ρ: 0.204<br>*p*-value: 0.108	ρ: 0.091<br>*p*-value: 0.479	ρ: 0.235<br>*p*-value: 0.064	**ρ: 0.258<br>*p*-value: 0.004**
**Legumes** *(n)*	**ρ: 0.272<br>*p*-value: 0.031**	ρ: -0.031<br>*p*-value: 0.809	ρ: 0.219<br>*p*-value: 0.085	ρ: 0.261<br>*p*-value: 0.039
**Vegetable oils** *(n)*	ρ: 0.239<br>*p*-value: 0.060	ρ: -0.020<br>*p*-value: 0.878	ρ: 0.243<br>*p*-value: 0.056	ρ: 0.173<br>*p*-value: 0.175
**Tea/coffee** *(n)*	ρ: 0.042<br>*p*-value: 0.738	ρ: 0.046<br>*p*-value: 0.719	ρ: 0.051<br>*p*-value: 0.693	ρ: 0.022<br>*p*-value: 0.865
**Fruit juices** *(n)*	ρ: -0.010<br>*p*-value: 0.947	ρ: 0.051<br>*p*-value: 0.696	ρ: 0.087<br>*p*-value: 0.503	ρ: -0.013<br>*p*-value: 0.919
**Refined grains** *(n)*	ρ: -0.016<br>*p*-value: 0.901	ρ: -0.055<br>*p*-value: 0.666	ρ: -0.004<br>*p*-value: 0.975	ρ: -0.142<br>*p*-value: 0.268
**Potatoes** *(n)*	ρ: 0.014<br>*p*-value: 0.912	ρ: -0.190<br>*p*-value: 0.135	ρ: 0.065<br>*p*-value: 0.613	ρ: -0.038<br>*p*-value: 0.770
**Sugar-sweetened beverages** *(n)*	ρ: 0.025<br>*p*-value: 0.875	ρ: 0.095<br>*p*-value: 0.458	ρ: 0.092<br>*p*-value: 0.474	ρ: -0.050<br>*p*-value: 0.696
**Sweet dessert** *(n)*	ρ: 0.102<br>*p*-value: 0.247	ρ: 0.078<br>*p*-value: 0.544	ρ: -0.088<br>*p*-value: 0.491	ρ: -0.004<br>*p*-value: 0.977
**Animal fat** *(n)*	ρ: -0.235<br>*p*-value: 0.064	ρ: 0.078<br>*p*-value: 0.544	ρ: -0.237<br>*p*-value: 0.062	**ρ: -0.319<br>*p*-value: 0.011**
**Dairy** *(n)*	**ρ: -0.327<br>*p*-value: 0.009**	ρ: 0.200<br>*p*-value: 0.116	**ρ: -0.280<br>*p*-value: 0.009**	**ρ: -0.406<br>*p*-value: 0.001**
**Egg** *(n)*	ρ: -0. 207<br>*p*-value: 0.103	ρ: 0.133<br>*p*-value: 0.297	ρ: -0.172<br>*p*-value: 0.177	ρ: -0.254<br>*p*-value: 0.044
**Fish/seafood** *(n)*	ρ: -0.202<br>*p*-value: 0.102	**ρ: 0.291<br>*p*-value: 0.020**	ρ: -0.183<br>*p*-value: 0.153	ρ: -0.188<br>*p*-value: 0.140
**Meat** *(n)*	**ρ: -0.420<br>*p*-value: 0.001**	ρ: 0.175<br>*p*-value: 0.171	**ρ: -0.400<br>*p*-value: 0.001**	**ρ: -0.447<br>*p*-value: <0.001**

**Misc. Animal based foods** *(n)*	**ρ: -0.291** **p-value: 0.021**	ρ: 0.041 p-value: 0.750	ρ: -0.260 p-value: 0.040	**ρ: -0.314** **p-value: 0.012**
**Plant milk** *(n)*	ρ: 0.116 p-value: 0.365	ρ: 0.132 p-value: 0.303	ρ: 0.162 p-value: 0.203	**ρ: 0.252** **p-value: 0.047**
**Plant yoghurt** *(n)*	**ρ: 0.307** **p-value: 0.015**	ρ: -0.101 p-value: 0.431	**ρ: 0.243** **p-value: 0.055**	**ρ: 0.307** **p-value: 0.015**
**Misc. plant-based foods** *(n)*	ρ: 0.130 p-value: 0.310	ρ: -0.243 p-value: 0.056	ρ: 0.165 p-value: 0.196	ρ: 0.091 p-value: 0.477

Supplementary Table 6 legend: SSB = Sugar-Sweetened Beverages. Misc- = Miscellaneous. Spearman's rank-order correlations based on observations of n = 63 participants. P-values <0.05 are shown in bold.

Supplementary Figures

Supplementary Figure 1

Supplementary Figure 1 title: Study scheme

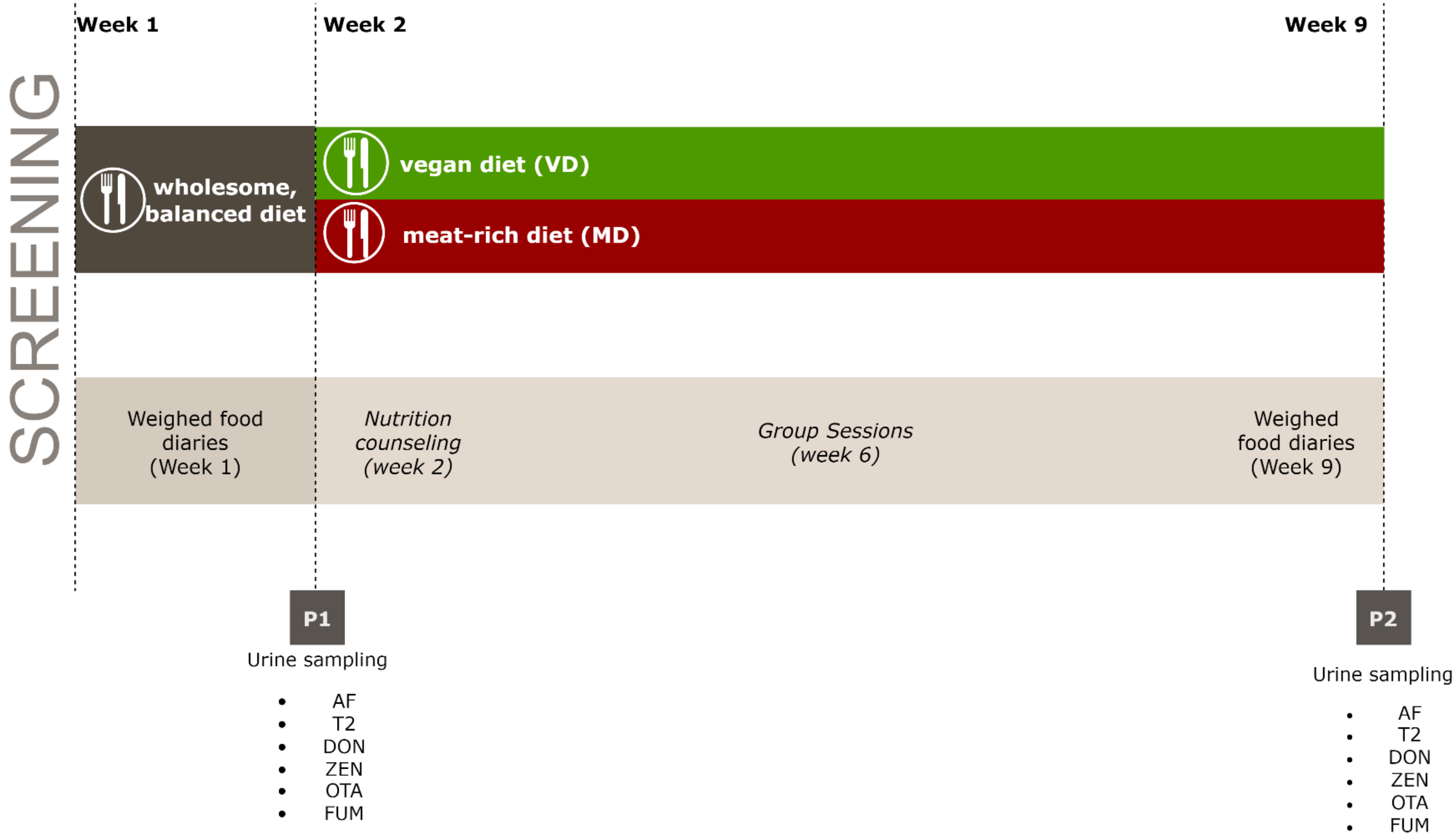

Supplementary Figure 1 legend: AF = Aflatoxin; DON = Deoxynivalenol; FUM = Fumonisin; OTA = Ochratoxin A; T2 = T-2 toxin; ZEN = Zearalenone

Supplementary Figure 2

Supplementary Figure 2 title: Participant inclusion flowchart

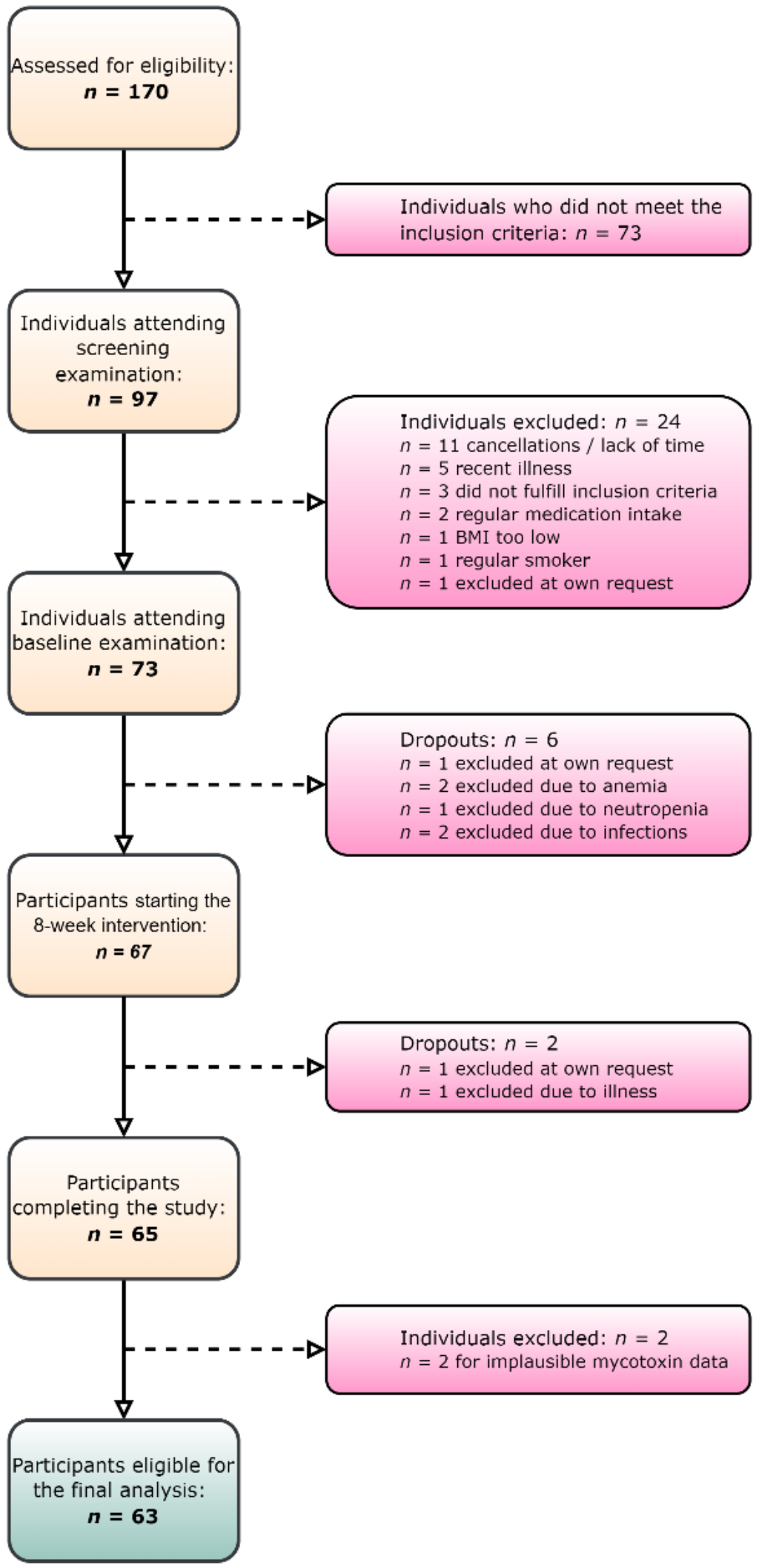

Supplementary Figure 1 legend: modified from [22][23]

Supplementary Figure 3

Supplementary Figure 3 title: Strip plot displaying aflatoxin excretion in urine at baseline (week 0) and after eight weeks into the study with a meat-rich diet (red color) or vegan diet (green color).

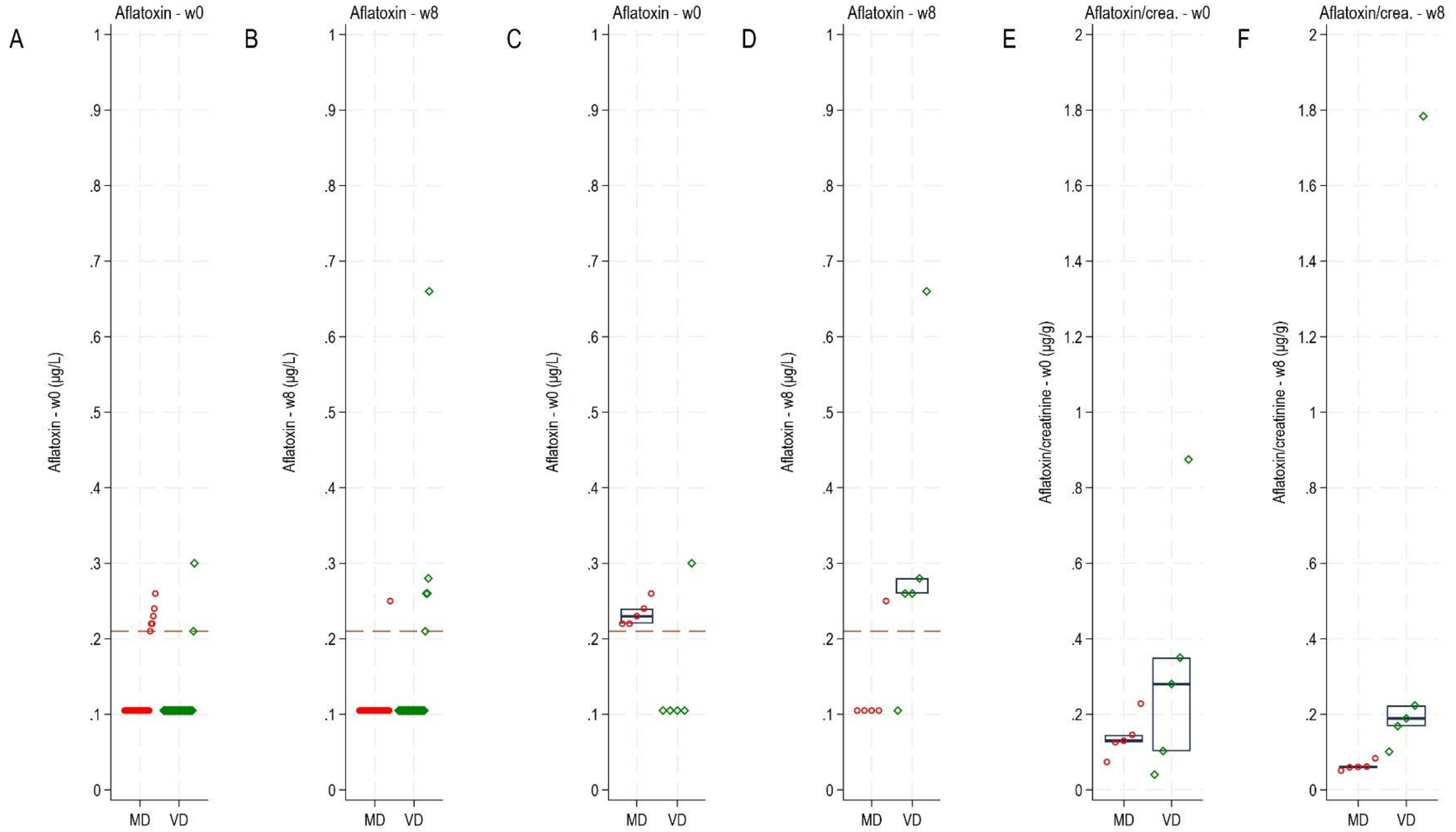

Supplementary Figure 3 legend: Panels A and B show the entire sample (including those observations below the detection limit) whereas panels C - F show only participants above the detection limit (0.21 µg/L). Panels E and F display creatinine-adjusted values (in µg/g). The horizontal dashed brown line marks the detection limit. MD = Meat-Rich Diet; VD = Vegan Diet. Aflatoxin concentrations were below the detection limit in *n* = 55 samples (87.3%) at baseline and *n* = 57 samples (90.48%) after eight weeks, respectively.

Supplementary Figure 4

Supplementary Figure 4 title: Scatter plots displaying spearman ranks of selected significant associations between creatinine-adjusted urinary mycotoxin excretion and food intakes at week 8 of the study – part I. Rank visualization suggests that a true monotonic relationship may only exist for vegetable intake and deoxynivalenol excretion as well as vegetable intake and ochratoxin A excretion.

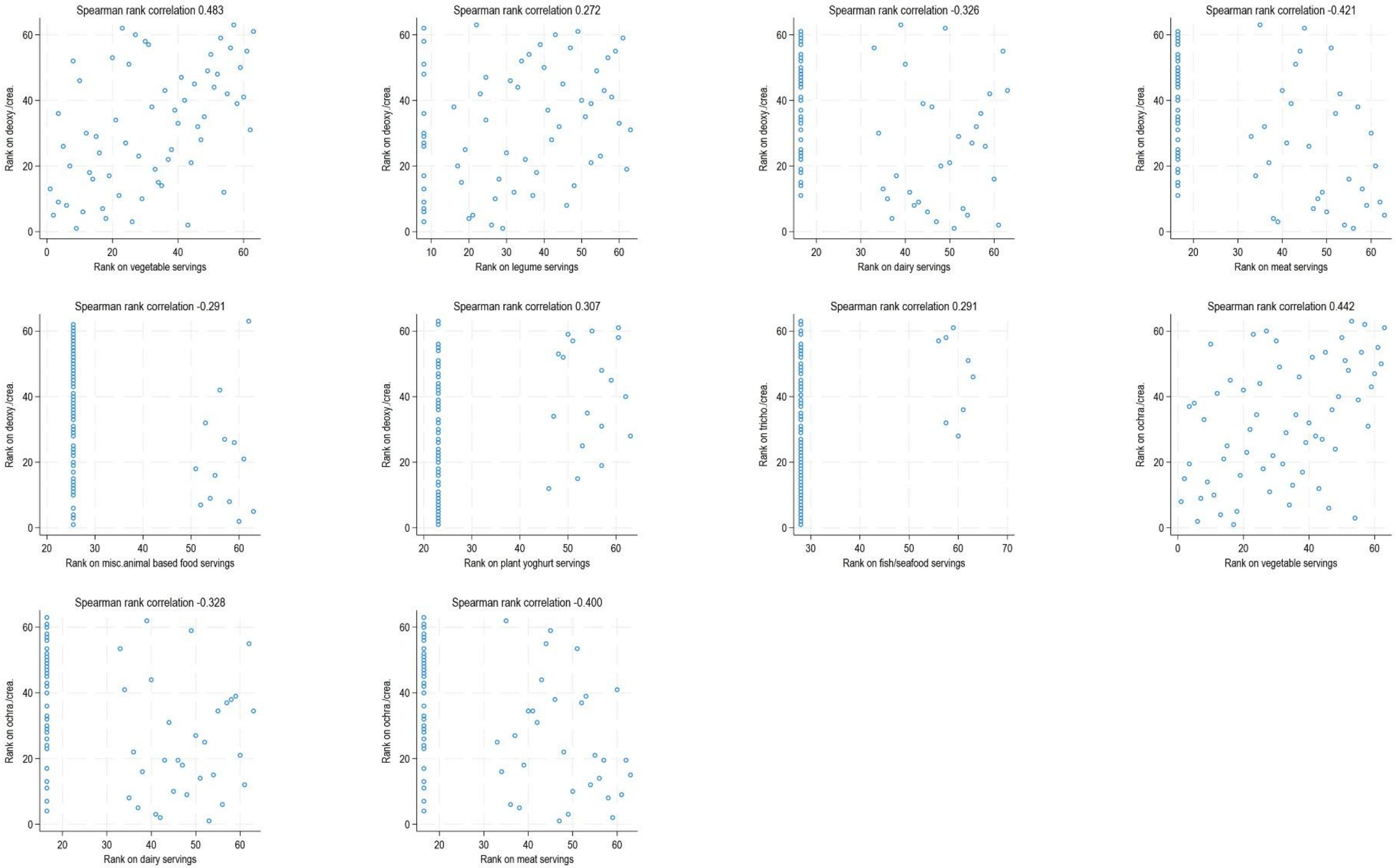

Supplementary Figure 5

Supplementary Figure 5 title: Scatter plots displaying spearman ranks of selected significant associations between urinary mycotoxin excretion and food intakes at week 8 of the study – part II. Rank visualization suggests that a true monotonic relationship may only exist for zearalenone excretion and whole grain intake.

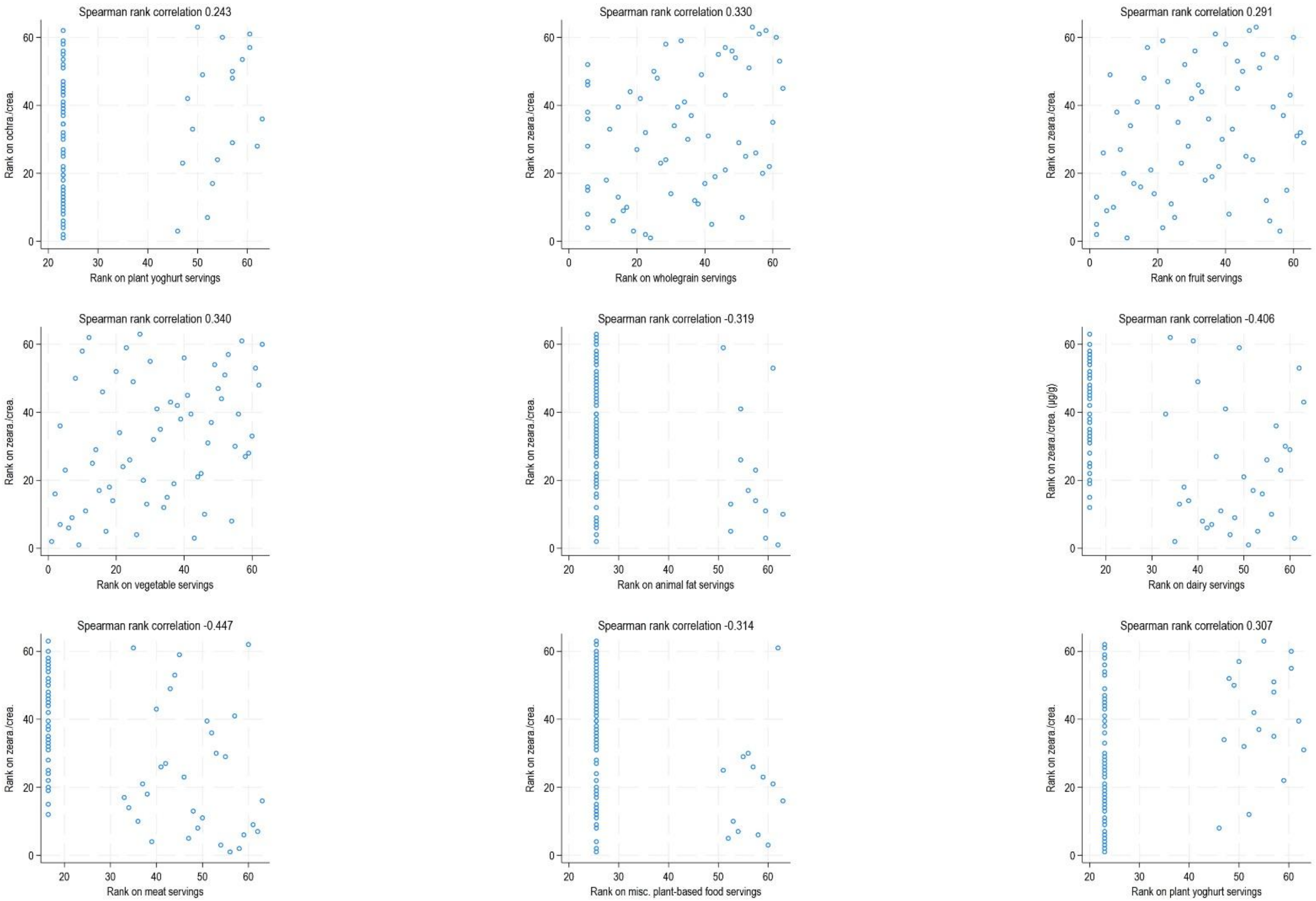

Supplementary Figure 6

Supplementary Figure 6 title: Restricted cubic spline regression models with 3 knots to potential model more complex, non-linear relationships between vegetable intake and creatinine-based mycotoxin excretion. Panels B and C suggested potential associations between vegetable intake and creatinine-adjusted deoxynivalenol and ochratoxin A excretion, respectively. Panel A suggested no significant association between vegetable intake and creatinine-adjusted zearalenone excretion. No convincing associations were found for all other food items (not shown).

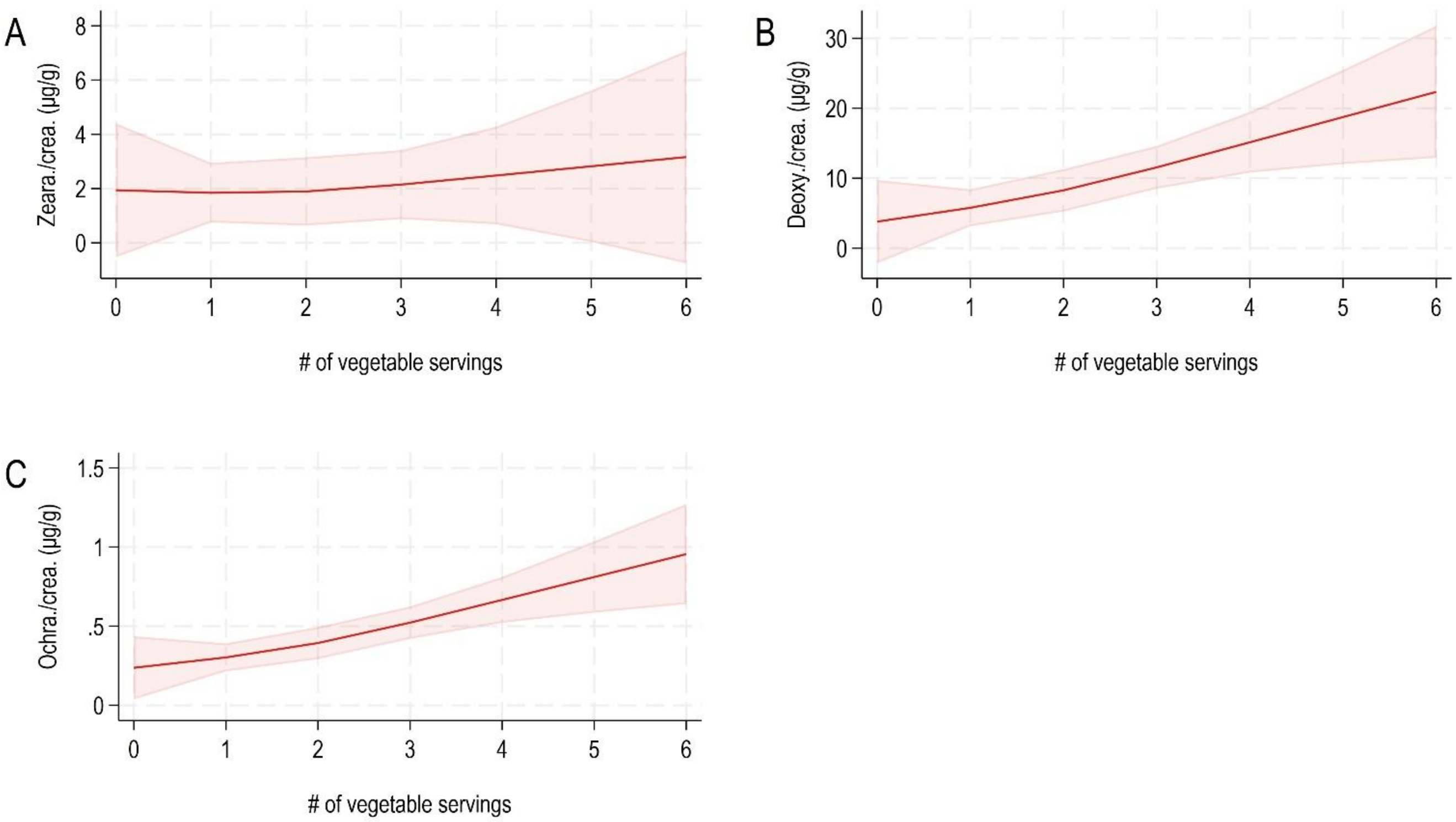

Supplementary Figure 7

Supplementary Figure 7 title: Energy intake (in kcal/day) at baseline (week 0) and at the end of the study (week 8) in the meat-rich diet group (MD, red color) and vegan diet group (VD, green color)

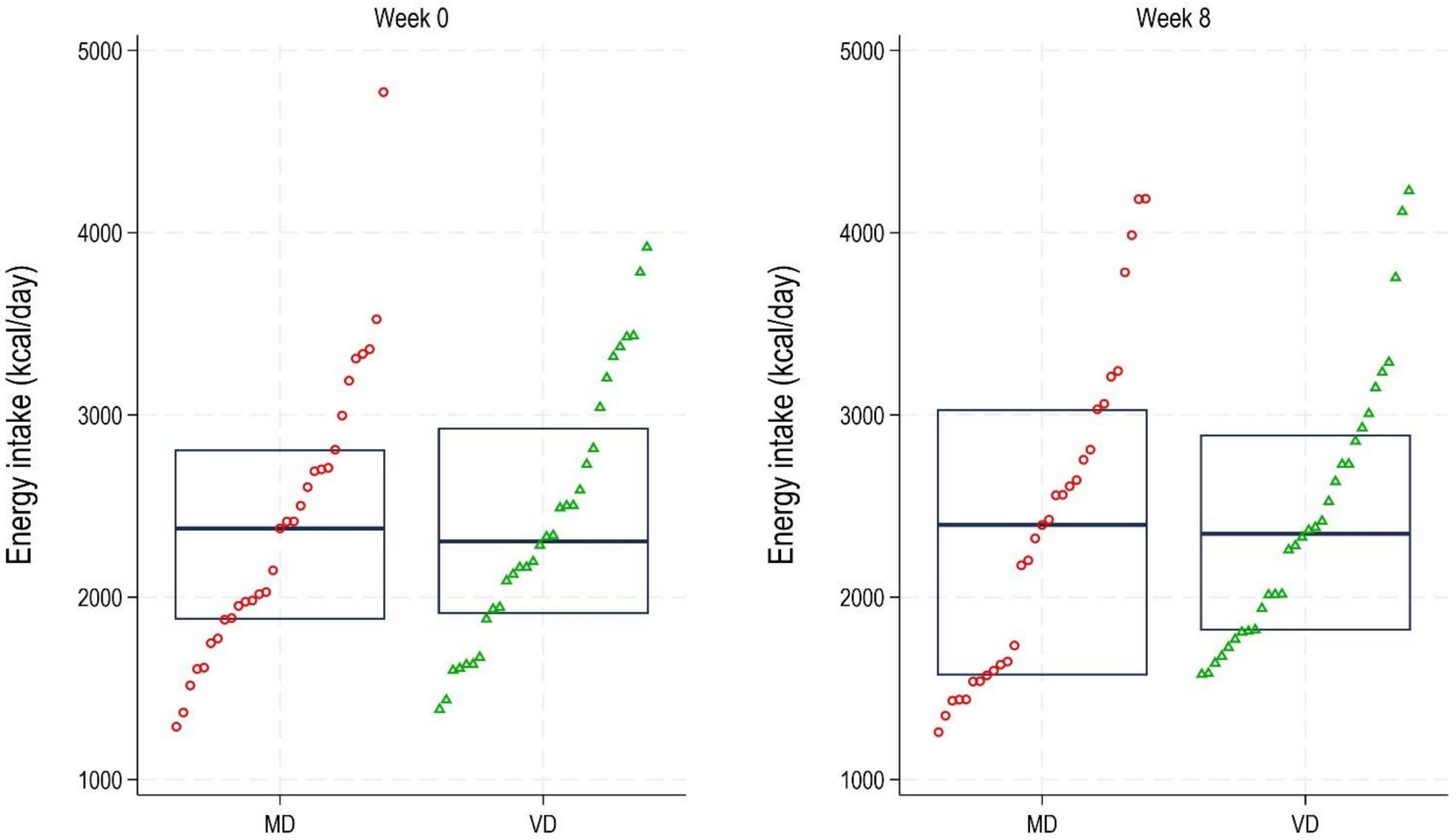

Supplementary Figure 8

Supplementary Figure 8 title: Urinary creatinine excretion (in g/L) at baseline (week 0) and at the end of the study (week 8) in the meat-rich diet group (MD, red color) and vegan diet group (VD, green color)

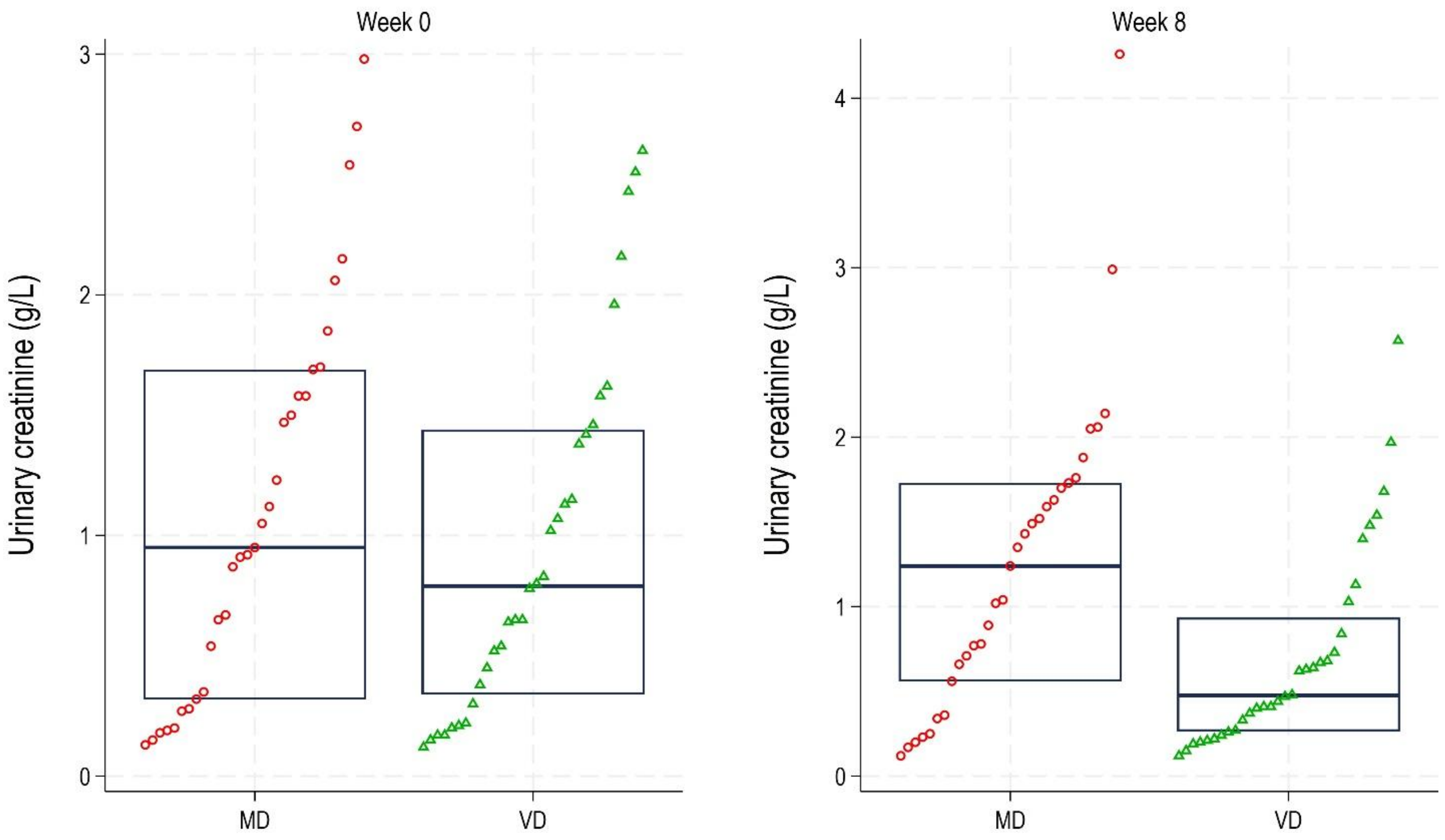

Supplement: Supplementary file 1 [file 41538_2026_1152_MOESM1_ESM.pdf]
